# Conformational analysis of nucleic acids for optimizing DNA and RNA topological models

**DOI:** 10.1093/nar/gkag668

**Published:** 2026-07-14

**Authors:** Philippe Archambault, Matthias Keil, Heidi M Muchall, Gilles H Peslherbe

**Affiliations:** Chemical Computing Group ULC, 910–1010 Sherbrooke W., Montréal, Québec H3A 2R7, Canada; Centre for Research in Molecular Modelling and Department of Chemistry and Biochemistry, Concordia University, 7141 Sherbrooke West, Montréal, Québec H4B 1R6, Canada; Chemical Computing Group ULC, 910–1010 Sherbrooke W., Montréal, Québec H3A 2R7, Canada; Centre for Research in Molecular Modelling and Department of Chemistry and Biochemistry, Concordia University, 7141 Sherbrooke West, Montréal, Québec H4B 1R6, Canada; Centre for Research in Molecular Modelling and Department of Chemistry and Biochemistry, Concordia University, 7141 Sherbrooke West, Montréal, Québec H4B 1R6, Canada

## Abstract

Conformational and configurational states of the sugar-phosphate backbone play an important role in DNA transcriptional and RNA translational events. In particular, the geometric parameters of the sugar ring dictate the degree of pucker, influencing the overall conformational state of the nucleic acid structure and its potential for base pairing and stacking. In this work, we explore nearly 50 years of nucleic acid structural data and compare over 4 million backbone torsion profiles of experimentally derived nucleic acid structures, and present a set of refined geometric parameters for nucleotides in common helix structures (A-, B-, and Z-helix). A comparison between X-ray and NMR structures for A- and B-helices underscores modelling challenges in accurately representing their conformational space. Furthermore, given the current interest in expanding the nucleoside library to include synthetic derivatives, these refined geometric parameters can serve in the development of conformational descriptors for analysing nucleic acid-based systems (e.g. small interfering RNA, microRNA, aptamers) with canonical and non-canonical base pairings.

## Graphical Abstract

**Graphical Abstract:**
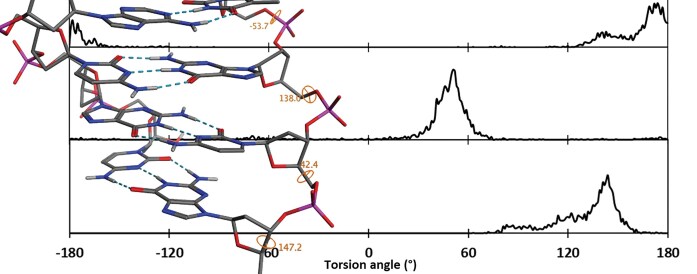

## Introduction

**Figure 1.**
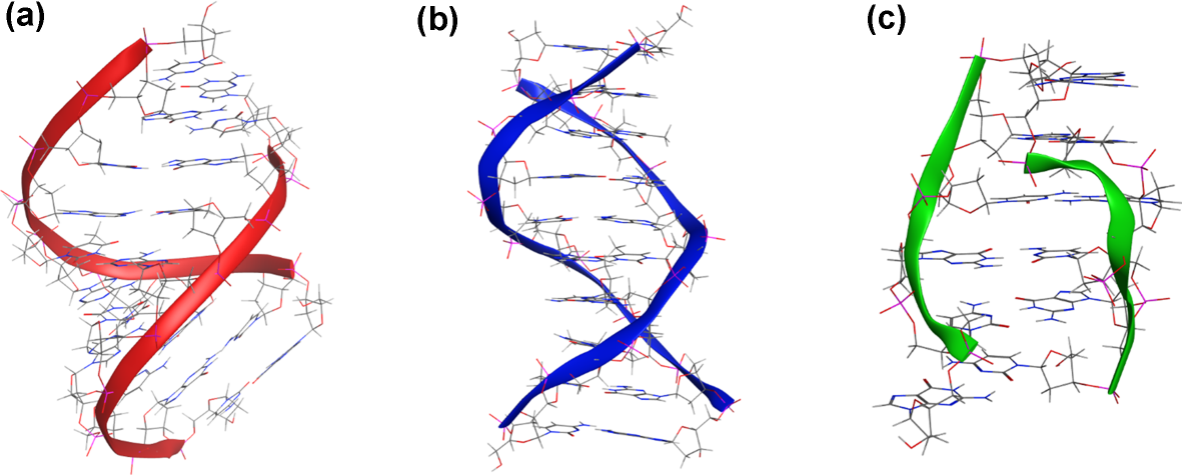
Examples of ultra-high-resolution structures with Watson–Crick base-paired nucleic acid helices obtained from the Research Collaboratory for Structural Bioinformatics (RCSB) [42, 43]. (**a**) PDB-ID: 1DPL [83] (A-helix DNA, resolution: 0.83 Å); (**b**) PDB-ID: 1D8G [84] (B-helix DNA, resolution: 0.74 Å); and (**c**) PDB-ID: 3P4J [85] (Z-helix DNA, resolution: 0.74 Å).

The structural intricacies of nucleic acids extend far beyond their canonical double-helix and single-stranded forms: secondary structures, tertiary interactions, and conformational dynamics play essential roles in cellular processes ranging from transcription and translation to complex regulatory mechanisms [1]. While structured RNA frequently adopts elaborate tertiary architectures through non-Watson–Crick interactions, these higher-order assemblies are constructed from recurring helical segments that provide the structural scaffold for loop, junction, and long-range interactions. Consistent with this hierarchical organization, approximately half of the nucleotides in experimentally determined nucleic acid structures participate in standard Watson–Crick double helices (Fig. 1) [2].

Additionally, geometric representation of the sugar-phosphate backbone is a prerequisite for interpreting, modelling, and validating nucleic acid structures across experimental and computational workflows. Although a vast number of DNA and RNA structures have been deposited over several decades, backbone torsional parameters are typically examined in isolation, derived from limited subsets of structures, or accessed through fragmented database interfaces. As a result, it remains difficult to assess the consistency, variability, and experimental context of backbone conformations across the full body of available structural data.

**Figure 2.**
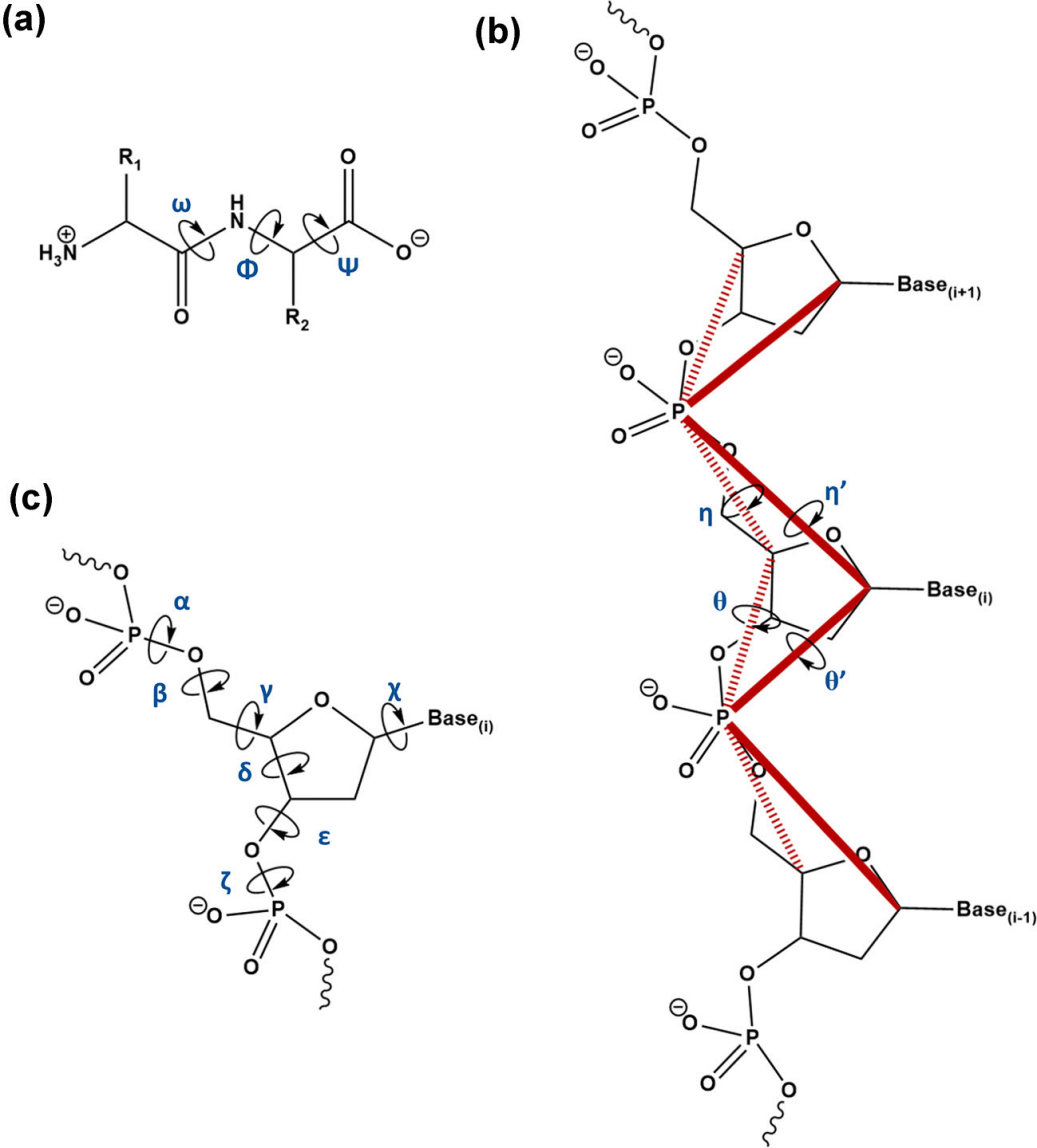
2D depiction of the backbone torsions and pseudotorsions found in proteins and nucleic acids. (**a**) Three peptide backbone torsions (Ψ, Φ, and ω); (**b**) six nucleotide backbone torsions (α, β, γ, δ, ε, and ζ) and glycosidic bond torsion (χ); and (**c**) two nucleotide pseudotorsions (η/η′ and θ/θ′).

Analysis of 2D plots of torsion angles has produced errors-based theories that appear extremely comprehensive as they list almost 15 000 distinct conformations [3]. However, such approaches can generate an unrealistic number of probable errors by flagging dihedral angles on every other residue. More recent rigorous statistical approaches have characterized over 120 unique local conformers of dinucleotides [4], and one may wonder if over-parameterization is present as simple general-purpose approaches, such as MolProbity [5–7], analyse all-atom contacts for steric clashes and have demonstrated real value in validating 3D structures of proteins, nucleic acids and complexes (i.e. protein–nucleic) [8–14]. Particular focus is placed on the sugar puckering, as nucleic acid residues will typically adopt either a C2′-endo or a C3′-endo puckering. Therefore, MolProbity performs an additional check on nucleic acid residues to verify the ε-torsion angle (Fig. 2 and Table 1) and sugar pucker, as defined by the base-phosphate distance, fall within specified ranges [5–7]. If the measured values are outside the specified allowed ranges, the residue is then flagged as an outlier with potentially having “incorrect” sugar puckering and/or backbone torsions.

**Table 1.** Atom definitions of biological backbone torsions^a^

Residue type	Torsion symbols	Torsion atoms^b^
Amino	ω	C_α (_*_i −_*_ 1)_-C _(_*_i −_*_ 1)_-N _(_*_i_*_)_- C_α (_*_i_*_)_
	Φ	C _(_*_i −_*_ 1)_-N _(_*_i_*_)_- C_α (_*_i_*_)_-C _(_*_i_*_)_
	Ψ	N _(_*_i_*_)_- C_α (_*_i_*_)_-C _(_*_i_*_)_-N _(_*_i _*_+ 1)_
Nucleic	α	O3′ _(_*_i −_*_ 1)_-P _(_*_i_*_)_-O5′ _(_*_i_*_)_-C5′ _(_*_i_*_)_
	β	P _(_*_i_*_)_-O5′ _(_*_i_*_)_-C5′ _(_*_i_*_)_-C4′ _(_*_i_*_)_
	γ	O5′ _(_*_i_*_)_-C5′ _(_*_i_*_)_-C4′ _(_*_i_*_)_-C3′ _(_*_i_*_)_
	δ	C5′ _(_*_i_*_)_-C4′ _(_*_i_*_)_-C3′ _(_*_i_*_)_-O3′ _(_*_i_*_)_
	ε	C4′ _(_*_i_*_)_-C3′ _(_*_i_*_)_-O3′ _(_*_i_*_)_-P _(_*_i _*_+ 1)_
	ζ	C3′ _(_*_i_*_)_-O3′ _(_*_i_*_)_-P _(_*_i _*_+ 1)_-O5′ _(_*_i _*_+ 1)_
	χ ^c^	O4′ _(_*_i_*_)_-C1′ _(_*_i_*_)_-[N1, N9] _(_*_i_*_)_-[C2, C4] _(_*_i_*_)_
	η	C4′ _(_*_i −_*_ 1)_-P _(_*_i_*_)_-C4′ _(_*_i_*_)_-P _(_*_i _*_+ 1)_
	θ	P _(_*_i_*_)_-C4′ _(_*_i_*_)_-P _(_*_i _*_+ 1)_-C4′ _(_*_i _*_+ 1)_
	η′	C1′ _(_*_i −_*_ 1)_-P _(_*_i_*_)_-C1′ _(_*_i_*_)_-P _(_*_i _*_+ 1)_
	θ′	P _(_*_i_*_)_-C1′ _(_*_i_*_)_-P _(_*_i _*_+ 1)_-C1′ _(_*_i _*_+ 1)_

^a^2D structure representation of backbone torsions in Fig. 2.

^b^Atom names for a given residue (*i*) as well as its previous neighbour (*i −* 1) and next neighbour (*i *+ 1) in the 5′–3′ direction for nucleic torsions and N-terminus to C-terminus for the amino torsions.

^c^χ-torsion atoms N1/C2 and atoms N9/C4 for pyrimidine and purines, respectively.

The source of the data can play a large role in the outcome of the analysis, with the two major techniques being X-ray crystallography and NMR spectroscopy for determining experimental structures. The process of crystallization imposes constraints on the available conformations due to the packing interactions between nucleic acid molecules (or in complexes with other species, such as proteins and ligands) [15, 16]. Understanding the impact of crystal packing is crucial for accurately interpreting structural data and distinguishing biologically relevant conformations from artifacts introduced during crystallization [17–21]. In some cases, crystal structures may capture conformations (artifacts) that are not representative of the *in vitro/in vivo* structures of nucleic acids [22]. Therefore, researchers must exercise caution in extrapolating functional insights solely from more rigid structures obtained from crystallography, as these structures may not adequately reflect physiological conditions. To capture the dynamic nature of nucleic acid structures, one should also consider incorporating solution behaviour observed in NMR experiments, while keeping in mind the limitations associated with NMR structures. These limitations include, but are not limited to, system size (typically <50 nt), the use of unphysiological salt concentrations, as well as computational challenges. For instance, NMR restraints (such as those from NOE experiments) provide boundaries for interactions and torsions for models fitted using force-field approximations [23, 24]. Overall, considering both the structured nature of crystal data and the flexibility observed in NMR structures presents a balanced approach to ensure a comprehensive assessment of the inherent structural characteristics.

A precise set of backbone parameters also is of importance in establishing a standardized baseline for nucleic acid conformational descriptors as they serve as the baseline, facilitating the accurate measurement of distortions induced by modifications or complexation (with small molecules, proteins, or other nucleic acid chains). This baseline is particularly relevant for modelling RNA therapeutics, such as antisense oligonucleotides and small interfering RNA (siRNA). Many of these systems incorporate chemical modifications not present in biological nucleic acids, including phosphorothioate (Rp/Sp), 2′-fluoro, and 2′-O-methyl substitutions. These modifications have been shown experimentally to preserve the overall helical geometry of RNA duplexes, as evidenced by circular dichroism spectra routinely reported in the literature [25, 26]. Within this context, a native-backbone conformational reference remains valuable for modelling and interpretation: deviations from canonical helical behaviour observed in simulations of novel or less characterized modifications may signal steric or electronic perturbations that merit further experimental investigation. For siRNA in particular, maintenance of helical stability is a critical design objective, and comparison against a well-defined native baseline provides a practical framework for assessing the structural impact of chemical modification [27, 28].

Hence, a return to the fundamentals begins with an accurate and revised representation of Watson–Crick base-paired helices. While distinct geometric parameters of the canonical double-helix structures have been previously reported [19, 29–41], new structures are constantly being added to the RCSB [42, 43] and curated into the Nucleic Acid Database (NDB) [44, 45]. Consequently, these parameters should be revisited.

Therefore, this work explores the culmination of over half a century’s worth of nucleic acid structural data while highlighting the consistency of backbone torsion angles across diverse secondary structures. Curated datasets of exclusively A-, B-, and Z-helix structures are created to present updated circular mean torsion values for the three common helix structures in the absence of proteins and small-molecule ligands. Comparative analyses are used to highlight the differences in torsion distributions between X-ray and NMR structures of A- and B-helix backbone geometry. Finally, this work addresses examples of the modelling associated with RNA therapeutics and small-molecule targeting, and the need for accurate references for developing structure-based descriptors.

## Methodology

To explore backbone torsions of nucleic acid structures, the NDB [44, 45] was searched for Protein Data Bank (PDB) IDs with experimentally determined structures. Each structure was downloaded from RCSB [42, 43] into a database for analysis using Molecular Operating Environment (MOE) [46]. Within the MOE DNA/RNA Builder, A-helix and B-helix conformations are available in two different variants. The first variants use the published parameters from Arnott and Hukins (A&H) [32], and the second variants (as well as the Z-helix conformation) use parameters provided by the w3DNA server [29, 33–35]. These helix conformation sources will serve as the references for comparison with the reported torsions in this work.

### Database of nucleic acid structures

Nine thousand nine hundred twenty-three unique PDB structures were investigated. The structures were obtained from three experimental techniques: (i) X-ray diffraction (X-ray; 8829 structures with resolutions ranging between 0.55 and 11.5 Å), (ii) neutron diffraction (ND; six structures with resolutions ranging from 1.40 to 3.0 Å), and (iii) NMR solution methods (NMR; 1086 structures).

While there are ‘ultra-high resolution’ structures (a resolution of 1 Å or less) of A-, B-, and Z-helical forms (examples in Fig. 1), relatively few are currently available, and selecting only these would have been too restrictive. Therefore, a more relaxed threshold of 2 Å or less was used, which corresponds to the previous threshold used by Olson and co-workers [36]. Structures that were co-crystalized with any ligands (small molecules) or protein chains were excluded, as well as nucleic acid chains of 5 or fewer residues. Using these criteria, 171 structures were evaluated as A-helix geometry, 135 structures as B-helix geometry, and 55 structures as Z-helix geometry in the X-ray dataset (Supplementary Table S1).

A major advantage of NMR-determined structures is that the ensemble of structures can provide information on their behaviour in solution. Recent work by Fowler and co-workers [24] demonstrates the accuracy of NMR protein structures in the RCSB [42, 43]. While the majority of structures have well-determined secondary structure, typically poorly resolved regions, such as loops, were determined to be too flexible with respect to the ‘real’ structure. The refinement of NMR structures relies heavily on the quality of geometric parameters and force fields. Therefore, geometrical tests can provide a good approximation for structure comparison but are not in themselves a single measurement of accuracy. Applying this outlook to nucleic acid systems, canonical helices can serve as a starting point to benchmark for ‘rigid’ nucleic acid conformations before investigating additional secondary structures such as more conformationally flexible loops. The additional data (conformational ensemble) provided by NMR-deposited structures can help to provide insight into the flexibility of the helix geometries and compare them to the more rigid X-ray-determined structures. Therefore, the full ensemble of NMR-determined structures were used. Using the same criteria as the X-ray dataset, 17 structures were evaluated as A-helix geometry, 205 structures as B-helix geometry, and 1 structure as Z-helix geometry in the NMR dataset (Supplementary Table S2).

### Identification of nucleic acid residues

To distinguish between residue types (amino, nucleic, and other), SMARTS pattern variation of SMILES [47] were used. Variations in the patterns were also used to capture nucleic residues of different terminal types (Polymer, Free, 5′-end, 3′-end, Free + PO_4_, 5′ + PO_4_) as well as DNA versus RNA nucleotides. The SMARTS patterns used are summarized in Supplementary Tables S3–S5. These SMARTS patterns recognize the backbone atoms of the nucleotides (including sugar) and not the nucleobase. Therefore, the full dataset contains a combination of natural nucleobases as well as non-natural nucleobases. In total, 4 184 581 nt make up the full database, and the distribution of measured backbone torsions is summarized in Supplementary Fig. S1. Further filtering based on the residue names found in the deposited PDB files was used (DA, DC, DG, DT, A, C, G, U) to ensure that the measured average torsions reported in Table 1 were of natural bases in the classified canonical structures.

### Grouping of helix structures for measured torsions

Each nucleic acid structure was classified in the external reference files from the NDB, which specifies annotations on their structure description, nucleic acid type, conformation type, and secondary structure [45]. This classification was used to create three groups of structures: (i) A-helix, (ii) B-helix, and (iii) Z-helix. A distinction between purine and pyrimidines in Z-helix has already been long established; pyrimidines adopt a *syn*-conformation and purines adopt an *anti*-conformation [19, 48]. Therefore, the Z-helix structures are investigated in two sub-groups: (i) Z (R) and (ii) Z (Y) for purines and pyrimidines, respectively.

The backbone atoms of canonical polynucleic acid chains are comprised of six single bonds whose torsions are denoted by α, β, γ, δ, ε, and ζ, represented in Fig. 2b along with χ, denoting the torsion of the N-glycosidic bond (all torsions defined in Table 1). When also considering sugar puckering, a polynucleic acid chain backbone has eight torsional degrees of freedom. This makes nucleic acid modelling, structure building, and prediction an exceptionally difficult challenge considering it is already not trivial for proteins, which have far fewer backbone torsions (Fig. 2a). Also explored was the common representation, or simplification, of polynucleic acid chain backbone geometry as defined by two sets of virtual torsions (pseudotorsions) η/η′ and θ/θ′ (Fig. 2c) to act as analogues to Ψ and Φ in proteins [39, 40]. However, due to the large similarities between the A- and B-helix geometries, the pseudotorsions alone offer less obvious distinctions between the two helix conformations. In contrast, Z-helix, unsurprisingly, being left-handed, does have significant differences in the pseudotorsions.

To assess the distribution of backbone torsions, the measured values were grouped into 1° bins, across the entirety of each torsion’s geometric range (−180° to +180°). Then, a range capturing the predominant peak was identified, and under the assumption of a normal distribution, the mean for that specific peak was determined. Due to the periodic nature of backbone torsions, the circular mean was used to ensure a more accurate representation of the circular distribution [49].

## Results and discussion

### Analysis of common nucleic acid helix backbone torsions

Nucleic acids systems, especially single-stranded RNA, make up a variety of different secondary structures. However, across 9923 structures and 4 184 581 nt, each backbone torsion remains relatively constant (Supplementary Fig. S1), in that the α-, β-, γ-, and ε-torsions demonstrate a single broad peak. Similarly, the χ-torsion does have such a dominant peak, but with a noticeable secondary peak, which highlights the broader χ-torsion range of the smaller pyrimidine nucleobases. The δ-torsion, as part of a ring system, is correlated with the ring puckering [37]. Its two distinct, sharper peaks can therefore easily be assigned to the more common C3′-endo/C2′-endo ring puckering. Most of the full dataset results from RNA structures, which adopt a C3′-endo puckering, and this is reflected in the distribution of the δ-torsions. While each torsion clearly has a distinct peak, each bin (field histograms are binned by 1°; −180° to +180°) is somewhat populated, making a Ramachandran-like plot with assigned allowed/not allowed torsion ranges less obvious.

In the following sections, we first discuss A- and B-helices with their more common helix geometries. This is followed by an analysis of Z-helical structures, which exhibit distinct conformational properties owing to their left-handed helical geometry. Ongoing work investigating additional secondary structures in greater detail will be devoted to a follow-up publication.

The geometric differences between the canonical double-helix structures (A- and B-helix) have long been known and characterized by many different parameters. Unsurprisingly, a specified structure will not always fit all the conventional parameters of A-, B-, or Z-helix geometry. As observed in the high-resolution X-ray dataset, the distribution of torsions spans broad ranges for each torsion. Nevertheless, as additional structures become available, the reported mean torsion values for polynucleic acid chains will gain increased reliability and precision, resulting in greater confidence in their accuracy.

### A-helix backbone geometry

**Figure 3.**
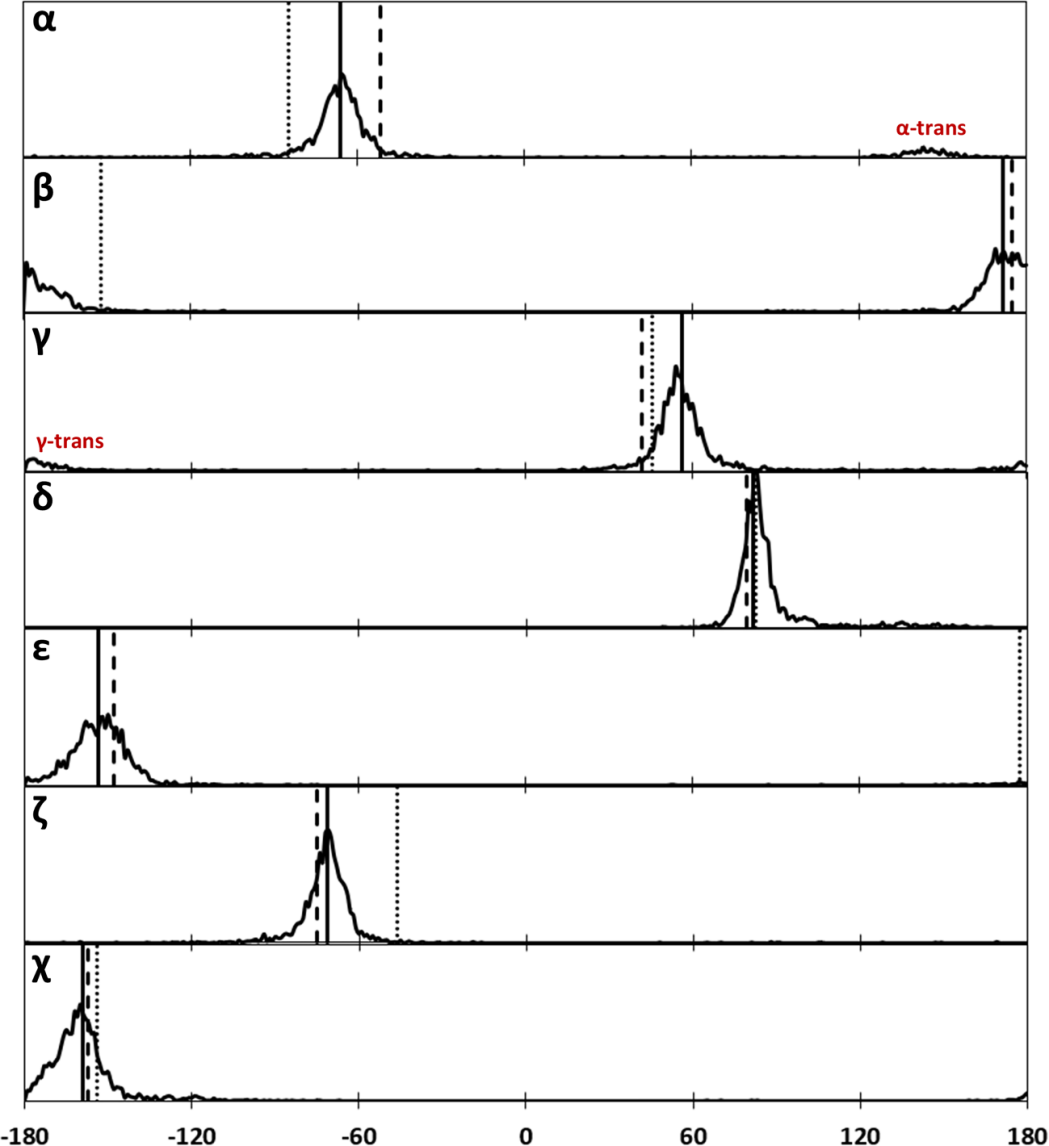
Field histogram of X-ray A-helix torsion angles (°). The *x*-axis is shared for each torsion and ranges from −180° to 180°, and the *y*-axis is the count of the frequency of occurrence by torsion angle (scale: 0–250). The solid vertical line represents the angle peak for each torsion measured in this work. The dashed vertical lines represent the values measured from the w3DNA server [29, 33, 34], and the dotted vertical line those measured by A&H [32]. Peaks associated with α-trans/γ-trans conformation are identified.

The measured circular means of the dominant peaks of the backbone torsions (Fig. 3 and Table 2) are in general agreement with previous publications of A-helix backbone torsions [19, 32, 33, 36]. In this work, the measured circular means from the X-ray dataset for the α-, β-, γ-, δ-, ε-, ζ-, and χ-torsions are at −66.2°, 171.7°, 56.1°, 81.6°, −153.2°, −71.4°, and −159.3°, respectively.

**Table 2.** Measured dominant torsion angles (°) for nucleic acid canonical helices^a^

Structure	Reference	α	β	γ	δ	ε	ζ	χ
A	A&H^b^	−84.7	−152.1	45.5	82.6	177.5	−46.3	−154.3
	w3DNA^c^	−51.7	174.8	41.7	79.1	−147.8	−75.1	−157.4
	This work^d^	−66.2	171.7	56.1	81.6	−153.2	−71.4	−159.3
B	A&H^b^	−46.9	−146.0	36.4	156.4	155.0	−95.1	−98.4
	w3DNA^c^	−29.9	136.4	31.1	143.4	−140.8	−160.5	−98.0
	This work^d^	−59.8	172.6	49.4	144.1	−172.6	−92.6	−106.7
Z (R)	w3DNA^c^	52.0	179.0	−173.8	94.9	−103.6	−64.8	58.7
	This work^d^	69.9	−172.5	176.5	92.2	−119.8	−69.3	63.6
Z (Y)	w3DNA^c^	−140.0	−137.0	51.0	138.0	−97.0	82.0	−159.6
	This work^d^	−153.9	−123.6	54.3	142.3	−95.0	76.6	−151.1

^a^A-helix, B-helix, and Z-helix (separated into purine [R] and pyrimidine [Y]).

^b^Arnott and Hukins [32].

^c^Measured from the w3DNA server ideal structures [29, 33–35].

^d^Torsions for this work are measured from the high-resolution X-ray dataset only.

The measured backbone torsions of the ‘ideal A-helix structure’ from w3DNA [33] are within 1.9°–14.5° (on average within 6.5°) of the circular mean measured from this high-resolution X-ray dataset. As expected, the δ-torsion has minimal deviation, within 1.9°, indicating its stability across many structures due to being part of the ribose/deoxyribose ring. The largest deviation, 14.5°, occurs with the α-torsion, followed closely by a 14.4° deviation with the γ-torsion. In general, the w3DNA A-helix backbone parameters closely resemble those of A-DNA fibre [19].

The reported backbone torsions of A-DNA from A&H [32] are within 1.0°–36.2° (on average within 18.0°) of the circular mean measured from the high-resolution X-ray dataset. The smallest deviation (1.0°) is expectedly the δ-torsion. The largest difference of 36.2° lies with the β-torsion, and the second largest of 29.3° with the ε-torsion. As more structures have become available in the last 50 years since A&H’s work [32], larger differences in the torsion values are not unexpected. It is important to note that despite the larger differences in the β-, ε-, and ζ-torsions, these backbone torsions still retained the general transoid conformation, making the reported values still similar in structure. From a modelling perspective, it would be quite likely that a minimization step to alleviate small van der Waals clashes (often referred to as simply ‘relaxing the system’) during structure preparation would result in very similar geometries.

**Figure 4.**
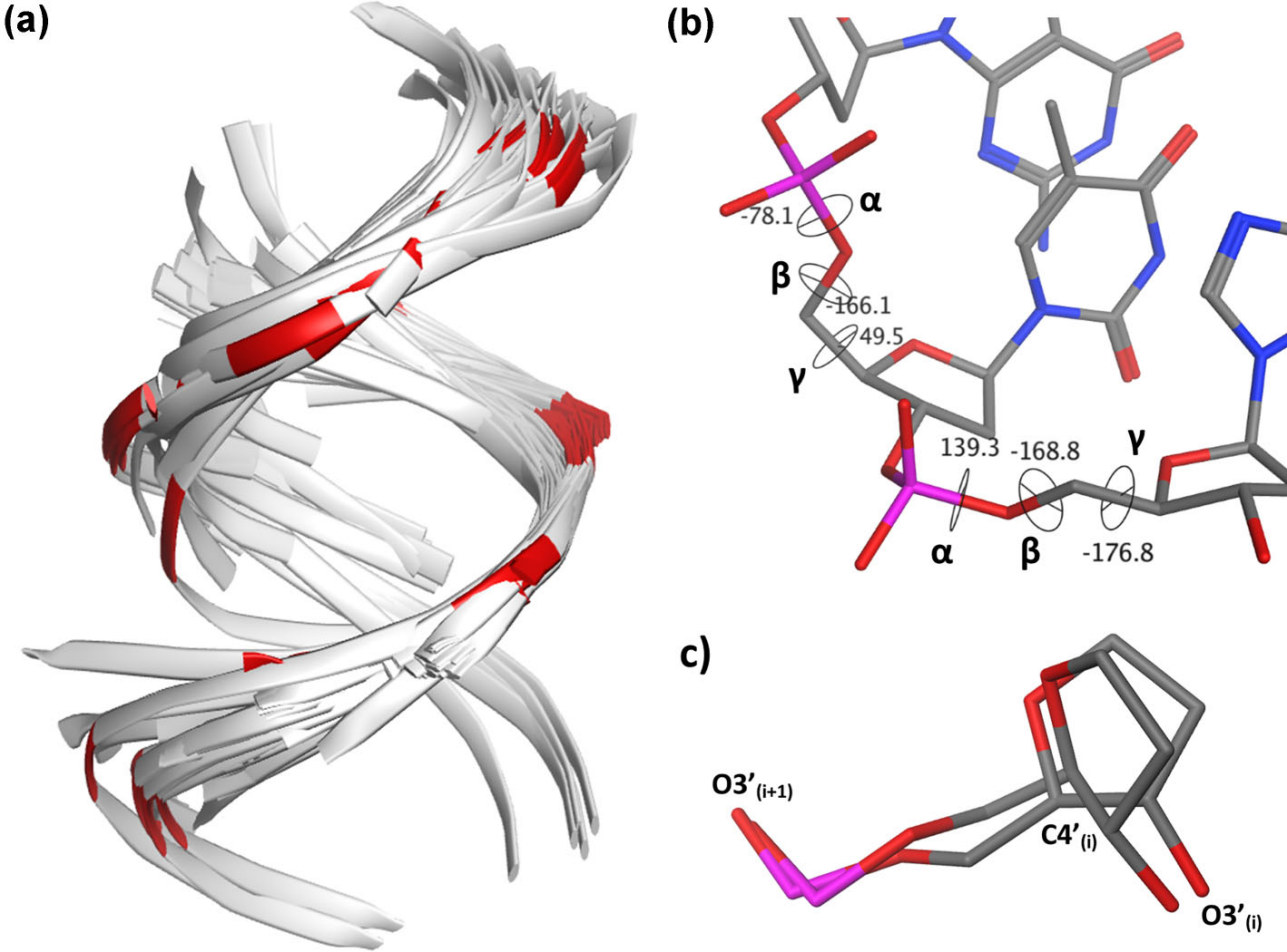
Selected A-helix structures with α-trans/γ-trans torsions. (**a**) Superposition of 131 A-helix X-ray structures that contain one or more secondary peak α-trans/γ-trans torsions. The helix backbone is represented by a grey ribbon, and the positions of the selected residues are given in red. (**b**) Two neighbouring residues showing the measured α-, β-, and γ-torsions. (**c**) Superposition of two residues at atoms O3′ _(_*_i _*_+ 1)_, C4′ _(_*_i_*_)_, O3′ _(_*_i_*_)_ (note that the hydrogen atoms as well as OP1, OP2, and nucleobase atoms are hidden for clarity).

A noticeable minor peak is present for both α- and γ-torsions and comprise ~9% of their measured torsions. These torsions (α-torsions ranging between +120° and +180° and γ-torsions ranging between +160° and −30°) are observed in 131 structures, or in almost 80% of the A-helix structures from the X-ray dataset. Structure superposition exposes that the changes in torsions occur at repeating positions along the helix (Fig. 4a). These torsion changes predominantly appear one to three times per chain, predominantly at *i *+* n* intervals (*n* = 0, 2, 4, 6). Examination of crystal packing indicates that these deviations arise at sites proximal to inter-helical crystal contacts (Supplementary Fig. S2), consistent with earlier evidence that crystal environment, rather than sequence, can influence the backbone conformation [18]. Notably, the altered torsions occur predominantly at purine residues, which occupy a larger steric volume and therefore offer less freedom for accommodating packing constraints. In contrast to proteins, nucleic acids cannot readily accommodate packing constraints through side-chain repacking without disrupting base stacking or secondary structure. Consequently, localized backbone rearrangements provide an efficient means of relieving steric and electrostatic strain imposed by crystal packing, particularly at sites where close backbone–backbone contacts would otherwise place negatively charged phosphate groups in unfavourable proximity.

At the time of publication of reference [18], only two high-resolution crystal structures of A-DNA (no ligand or proteins present within the crystal lattice) were available (PDB IDs: 295D [50] and 118D [51]), and it was noted that the altered torsions would occur in orthorhombic crystals but not hexagonal crystals. However, with additional high-quality structures now available, it is evident that the α-*trans*/γ-*trans* conformation can occur in various crystal systems, not just orthorhombic ones. The complete list of PDB entries exhibiting α-trans/γ-trans conformations, together with their corresponding space groups and crystal systems, is provided in Supplementary Table S6.

Overall, the presence of the coupled α-*trans*/γ-*trans* conformations does not impact the global structure, as the remaining atom positions of the phosphate backbone adjust (Fig. 4b) to maintain the helicity. A superposition of backbone atoms O3′ _(_*_i _*_+ 1)_, C4′ _(_*_i_*_)_, O3′ _(_*_i_*_)_ has a small RMSD of ~0.4 Å. Therefore, there are enough torsional degrees of freedom in the backbone of polynucleic acid chains that will not impact the overall structure. It is notable that while the deviations may mainly be attributed to crystal packing, they have been considered as targeting opportunities if they remain present in solution [52]. Furthermore, the α/γ transition, and similarly the ε/ζ transitions, highlight the inherent flexibility in the backbone [53, 54]. This flexibility is thought to accommodate nucleotide-flipping enzymes into adopting a more suitable backbone conformation for enzymatic activity [55].

### B-helix backbone geometry

**Figure 5.**
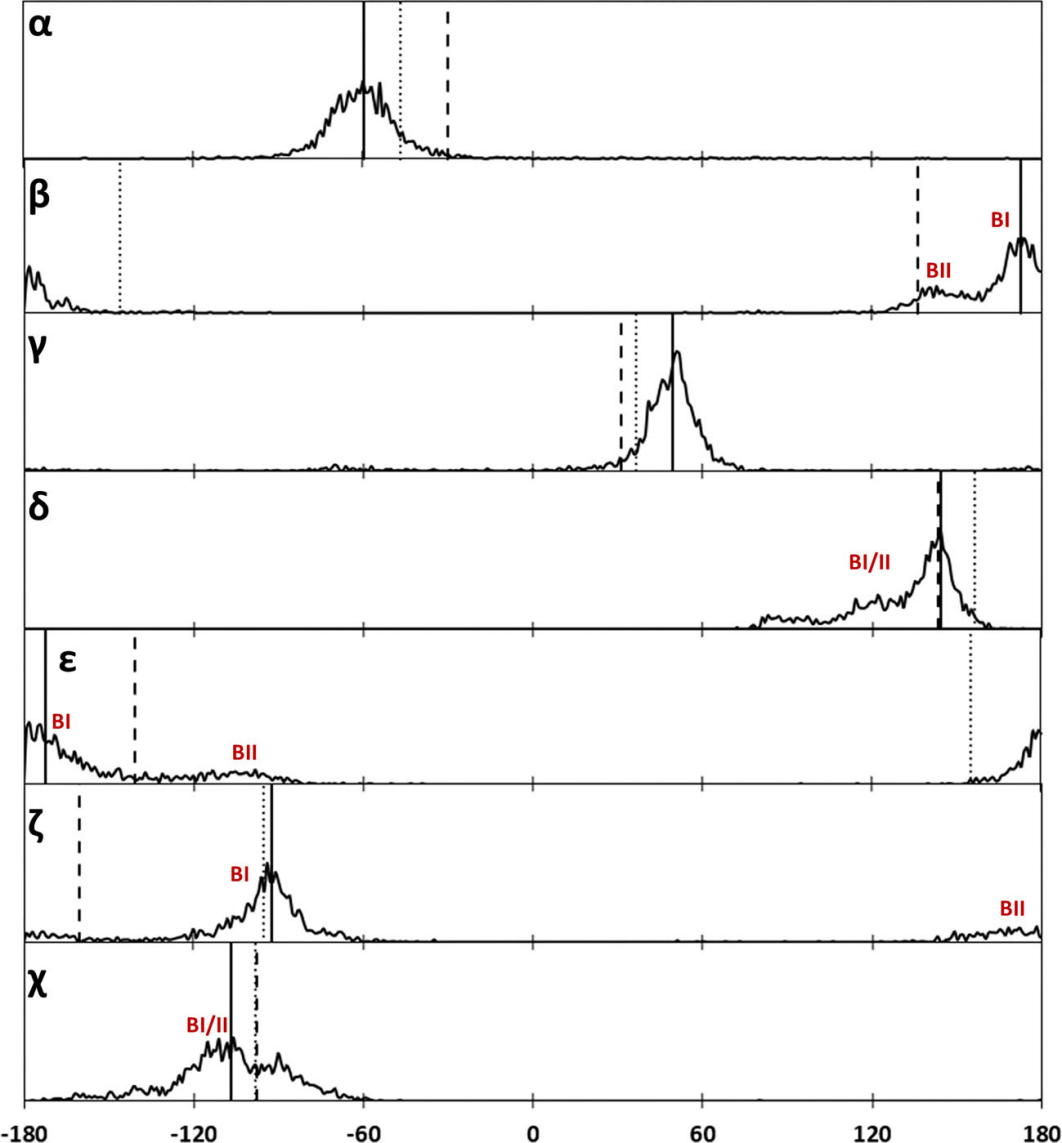
Field histogram of X-ray B-helix torsion angles (°). The *x*-axis is shared for each torsion and ranges from −180° to 180°, and the *y*-axis is the count of the frequency of occurrence by torsion angle (scale: 0–200). The solid vertical line represents the angle peak for each torsion measured in this work. The dashed vertical lines represent the values measured from the w3DNA server [29, 33, 34], and the dotted vertical line, those measured by A&H [32]. Peaks associated with BI-/BII-DNA are identified.

The measured circular means of the dominant peaks of the backbone torsions (Fig. 5 and Table 2) are in general agreement with previous publications of B-helix backbone torsions [19, 32, 33, 35]. In this work, the measured circular means from the X-ray dataset for the α-, β-, γ-, δ-, ε-, ζ-, and χ-torsions are at −59.8°, 172.6°, 49.4°, 144.1°, −172.6°, −92.6°, and −106.7°, respectively.

The measured backbone torsions of the ‘ideal B-helix structure’ from w3DNA [33] are within 0.7°–67.9° (on average within 27.6°) of the circular mean measured from the high-resolution X-ray dataset. As expected, the δ-torsion has minimal deviation (0.7°). The largest deviation, 67.9°, occurs with the ζ-torsion, followed by a 36.2° deviation with the β-torsion. In general, the w3DNA B-helix backbone parameters largely resemble that of B-DNA fibre [19].

The reported backbone torsions of B-DNA from A&H [32] are within 2.5°–41.4° (on average within 17.5°) of the circular mean measured from the high-resolution X-ray dataset. The smallest deviation (2.5°) is again the δ-torsion. The largest deviation, 41.4°, occurs with the β-torsion, followed by a 32.4° deviation with the ε-torsion. However, excluding these two torsions, the reported backbone torsions are on average within 10°. Unpredictably, A&H’s reported values [32] have on average smaller deviations with this work than the more recent w3DNA backbone parameters for the B-helix geometry.

In contrast to A-helix structures, there is a noticeable absence of a minor peak for α- and γ-torsions for B-helix (X-ray dataset; Fig. 5). Bingman and co-workers speculated that the α-*trans*/γ-*trans* conformation might be more prominent due to the C2′-endo ring being less rigid than the A-DNA C3′-endo counterpart [51]. While this does not appear to be the case for the α- and γ-torsions, the distributions of the remaining B-helix backbone torsions exhibit a significantly broader range with further apparent minor peaks.

It is well known that B-DNA is more flexible than A-DNA [4, 19, 51, 56–59] and that it has two major conformers: BI-DNA (more common) and BII-DNA [56, 57]. Some of the torsion differences [59] noted in the β- and ε-torsions appear as minor peaks in the distributions (Fig. 5). Additionally, while both conformers exhibit C2′-endo sugar puckering, evident from a dominant central peak, the δ-torsion shows a broad range of values with a few low-resolution maxima. For torsions where BI and BII conformations differ, the circular mean is less informative than the distinct BI and BII peaks. Calculating separate circular means for BI and BII would require clear separation of the two states, which may be difficult if the transition is gradual and demands additional considerations, such as state classification criteria or clustering approaches.

**Figure 6.**
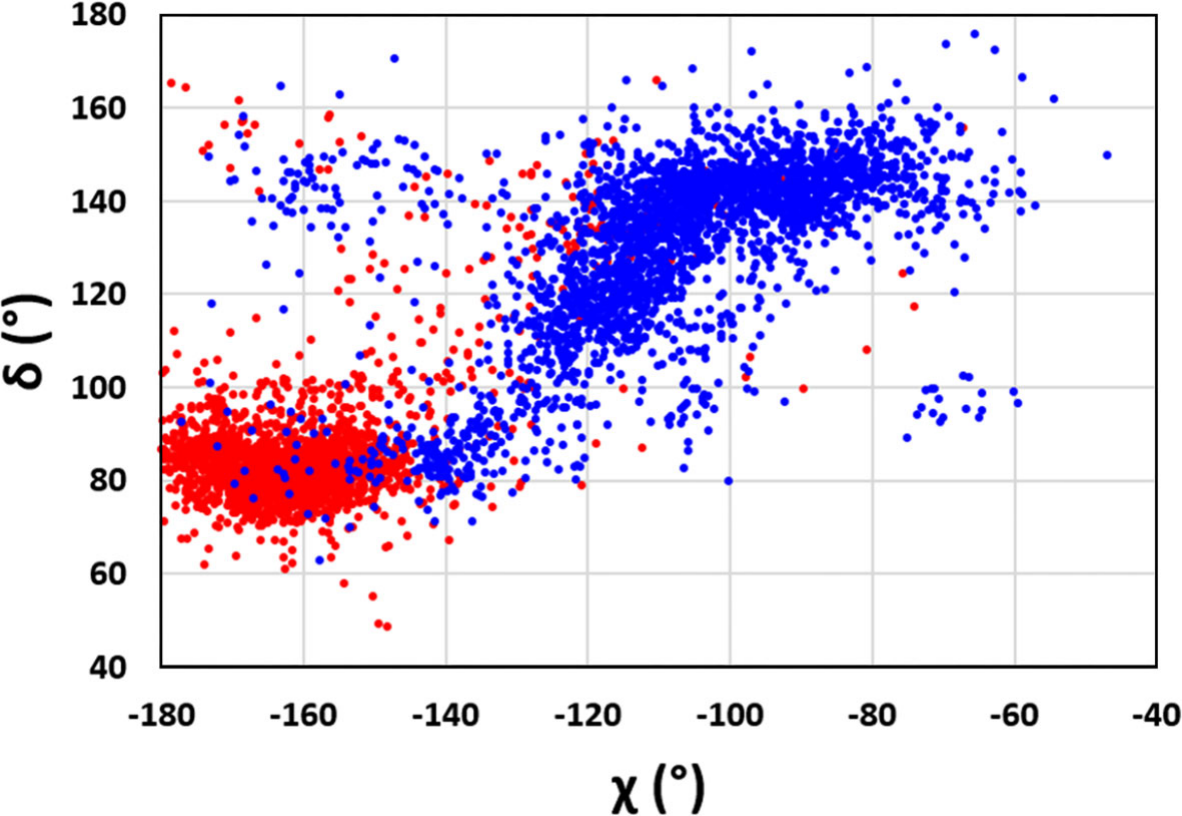
Scatter plot of nucleic acid δ/χ torsions. A-helix (red) and B-helix (blue) helices from X-ray diffraction methods with a resolution of 2 Å or less obtained from the RCSB [42,43] were used.

Complexation and environmental changes of DNA can lead to interconversion between A- and B-like forms in both the sugar ring and glycosyl rotation. Consistent with previous work by Lu and co-workers [36], the δ/χ coupling [31] distinguishes A- and B-DNA conformations (Fig. 6). However, the present dataset shows substantially greater overlap between the two populations than previously reported.

This increased overlap may reflect the larger number of available B-DNA structures, which sample a broader conformational space due to their greater intrinsic flexibility. As a result, B-DNA δ/χ values extend into regions typically associated with A-DNA. While this could suggest that some structures occupy geometries closer to easier A→B/B→A transitions pathways, such interconversion is not directly supported by the corresponding sugar puckering amplitudes.

These observations therefore indicate that overlap in δ/χ space alone should not be interpreted as evidence of conformational interconversion, but rather as a consequence of increased sampling of flexible B-DNA conformations.

### Z-helix backbone geometry

**Figure 7.**
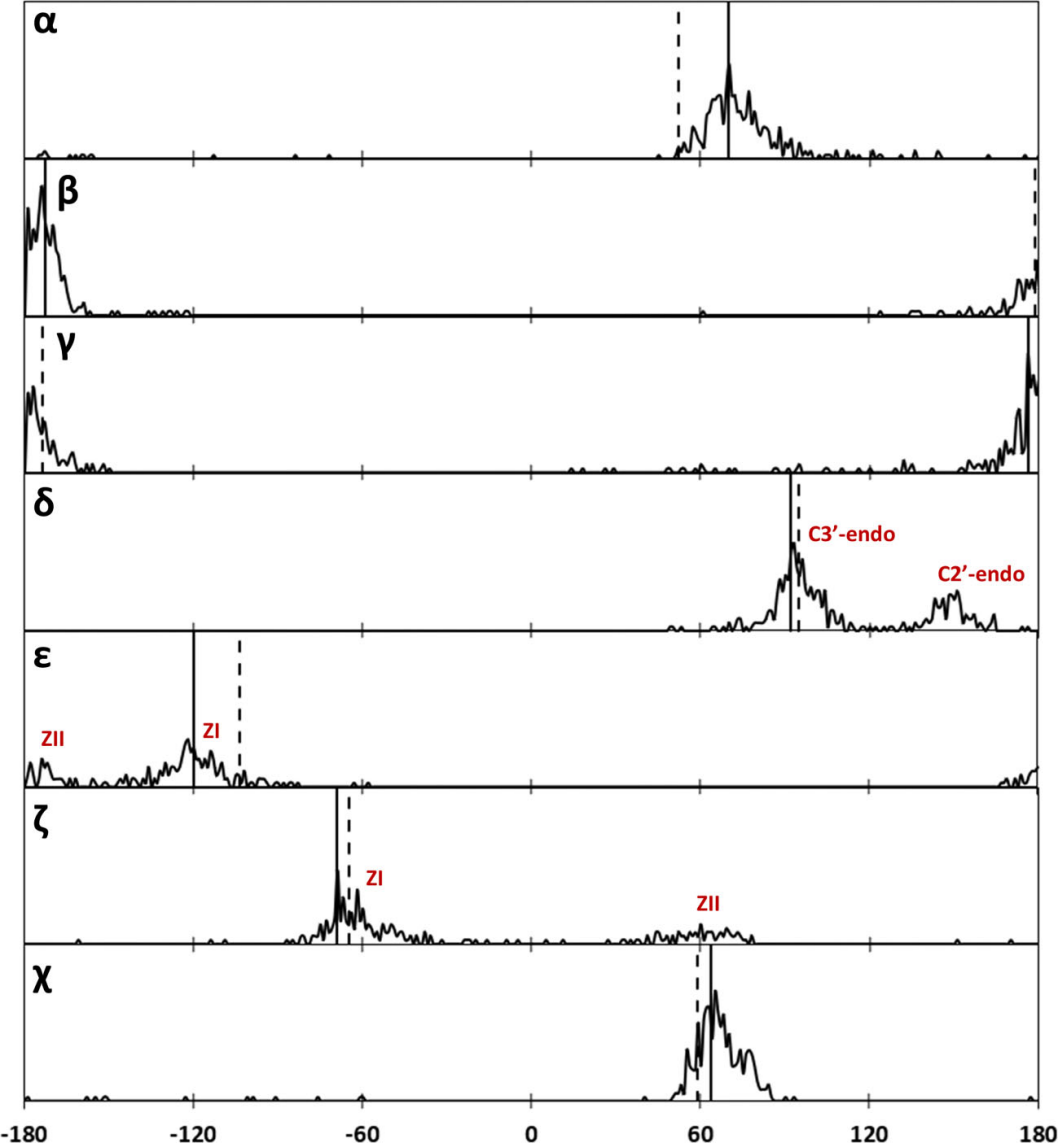
Field histogram of X-ray Z-helix (purine nucleotides) torsion angles (°). The *x*-axis is shared for each torsion and ranges from −180° to 180°, and the *y*-axis is the count of the frequency of occurrence by torsion angle (scale: 0–50). The solid vertical line represents the angle peak for each torsion measured in this work. The dashed vertical lines represent the values measured from the w3DNA server [29, 33, 34]. Peaks associated with ZI-/ZII-helix as well as C2′-/C3′-endo sugar puckering are identified.

**Figure 8.**
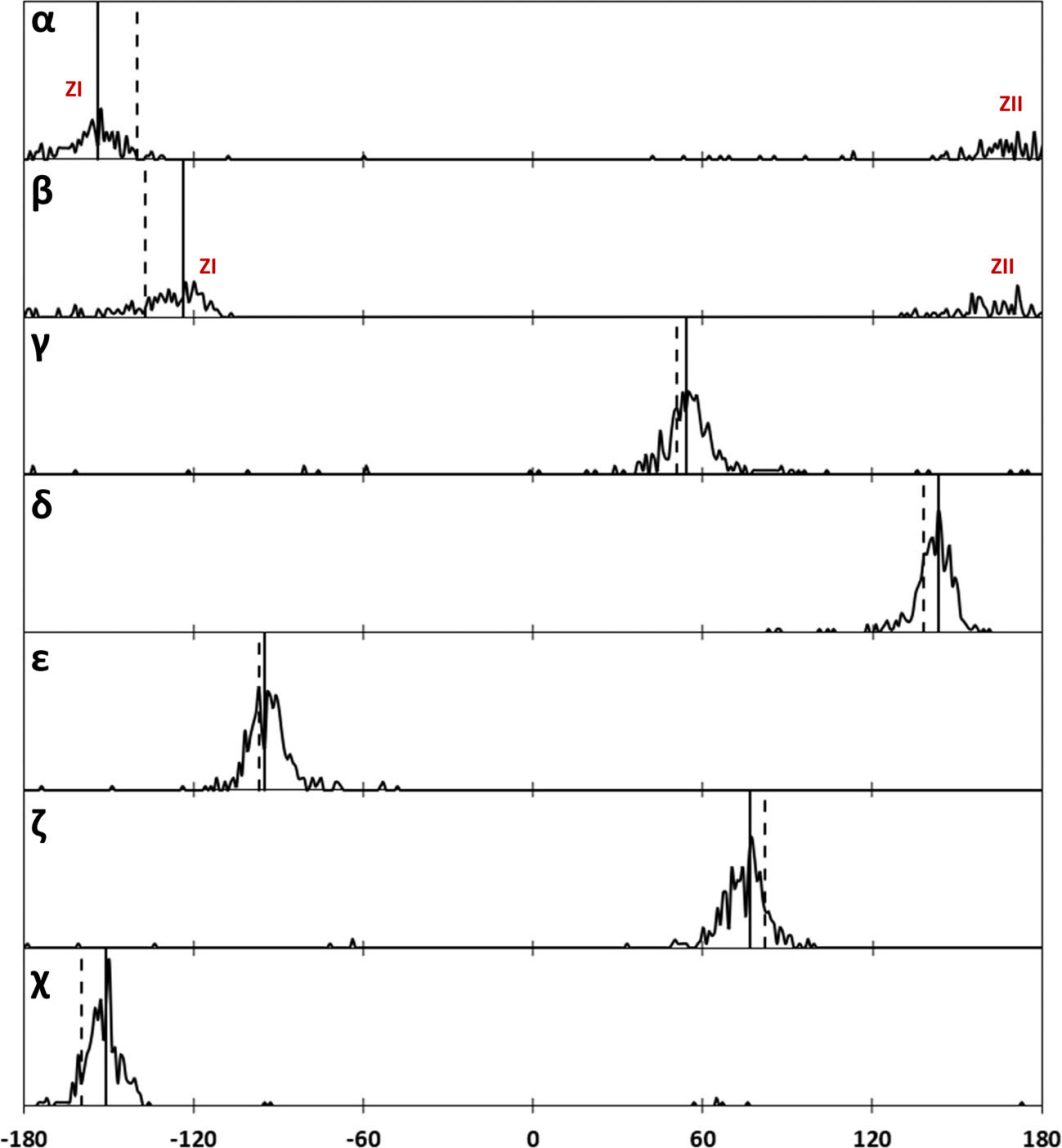
Field histogram of X-ray Z-helix (pyrimidine nucleotides) torsion angles (°). The *x*-axis is shared for each torsion and ranges from −180° to 180°, and the *y*-axis is the count of the frequency of occurrence by torsion angle (scale: 0–50). The solid vertical line represents the angle peak for each torsion measured in this work. The dashed vertical lines represent the values measured from the w3DNA server [29, 33, 34]. Peaks associated with ZI-/ZII-helix are identified.

The measured circular means of the dominant peaks of the backbone torsions (Figs 7 and 8; Table 2) are in general agreement with previous publications of Z-helix backbone torsions [19, 33]. In this work, the measured circular means from the X-ray dataset for the α-, β-, γ-, δ-, ε-, ζ-, and χ-torsions are at 69.9°, −172.5°, 176.5°, 92.2°, −119.8°, −69.3°, and 63.6°, respectively, for purines. In this work, the measured circular means from the X-ray dataset for the α-, β-, γ-, δ-, ε-, ζ-, and χ-torsions are at −153.9°, −123.6°, 54.3°, 142.3°, −95.0°, 76.6°, and −151.1°, respectively for pyrimidines.

The measured backbone torsions of the ‘ideal Z-helix structure’ from w3DNA [33] are within 2.7°–17.9° (on average 9.2°) and within 2.0°–13.9° (on average 7.4°) of the circular mean measured from the high-resolution X-ray dataset for purine and pyrimidine nucleobases, respectively. The smallest deviations are the δ-torsion (2.0°) for purine nucleobases and the ε-torsion (2.7°) for pyrimidine nucleobases. The largest deviation is in the α-torsion for both purine and pyrimidine nucleobases (17.9° and 13.9°, respectively).

Similar to B-DNA, there are two major conformers of Z-DNA: ZI-DNA and ZII-DNA. The two groupings can be distinguished in the ε- and ζ-torsion values for purines as well as in the α- and β-torsion values for pyrimidines. The measured distributions (Figs 7 and 8) support earlier work that determined that the ZI-DNA is populated approximately three times more than ZII-DNA [31, 48, 60]. A minor peak is also present for the purine group of Z-DNA, which is not typical for both ZI/ZII-DNA. This peak is predominantly attributed to residues at the end of chains, where more flexibility and, consequently, a broader distribution of ranges would be expected.

### Differentiation of common helix structures using pseudotorsions

**Figure 9.**
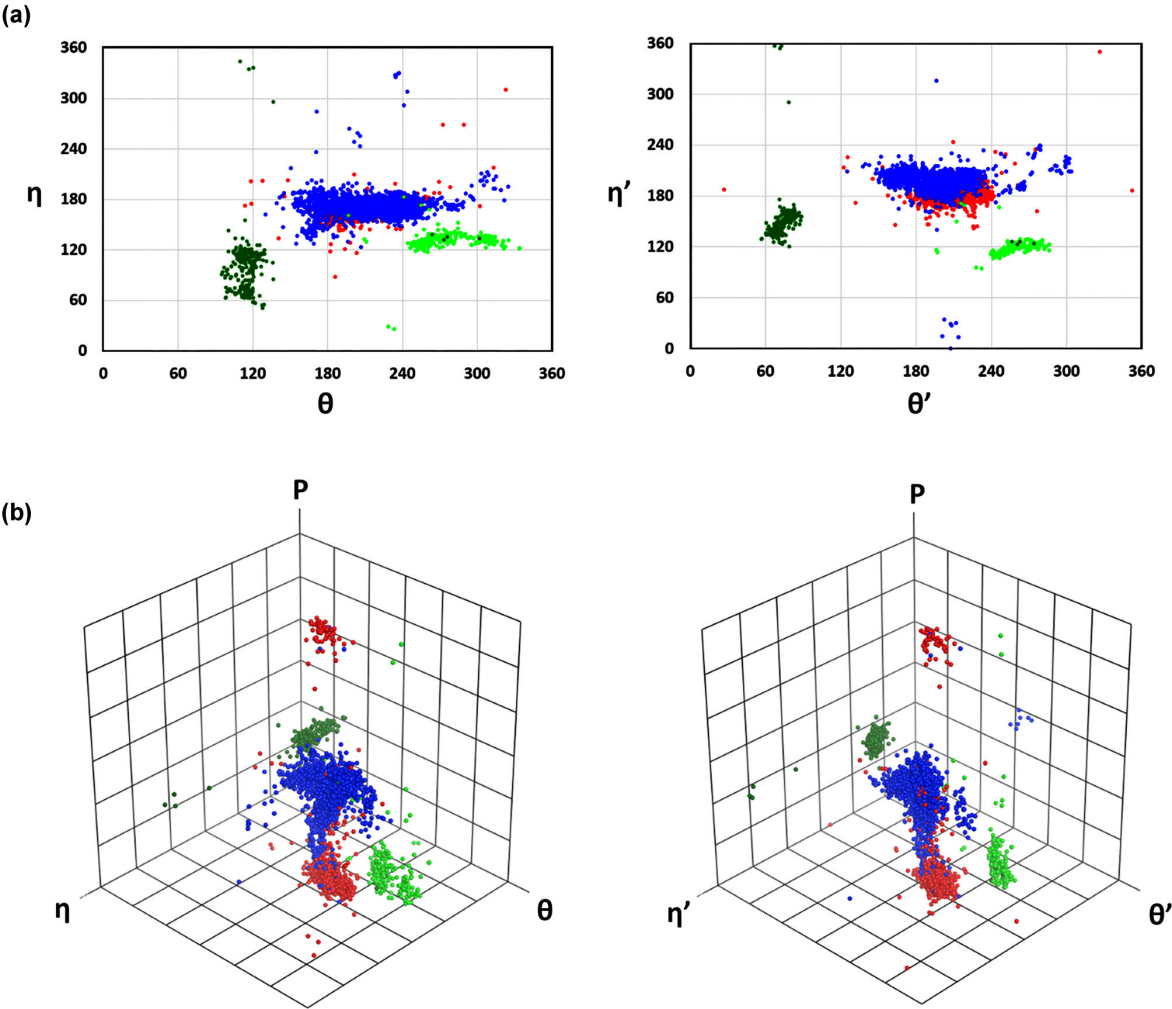
Scatter plots of nucleic acid virtual backbone parameters (pseudotorsions, η/θ and η′/θ′, and pseudorotation, P). DNA/RNA helices A-helix (red), B-helix (blue), and Z-helix (separated into purine and pyrimidine; green and dark green, respectively) from X-ray diffraction methods (resolution: 2 Å or less) obtained from the RCSB [42,43]. The x-, y-, and z-axes are from 0° to 360°. (**a**) 2D scatter plot of η/θ (left) and η’/θ’ (right). (**b**) 3D plot of η/θ/puckering (left) and η’/θ’/puckering (right).

Pyle and co-workers demonstrate distinct features of η–θ/η′–θ′ representations across a wide range of RNA structures [39, 40, 61]. Given their success in distinguishing diverse RNA secondary and tertiary structures, these reduced descriptors were examined for their ability to resolve more subtle conformational differences between canonical A-/B-helices. As expected, both being right-handed helices, they share similar pseudotorsions and are not readily distinguishable in η–θ/η′–θ′ plots (Fig. 9a), underscoring the risk of over-reliance on these reduced descriptors. In contrast, the left-handed helix (Z-helix) stands out as visibly unique clusters of purine and pyrimidine groupings. Incorporation of sugar puckering *via* measured pseudorotation provides an additional structural dimension that separates the overlapping A- and B-form populations (Fig. 9b).

While this distinction is well established for idealized helices, interpretation of experimental nucleic acid structures also benefits from the use of complementary descriptors. In practice, sugar pucker assignments can be uncertain, particularly at moderate resolution, and alternative pucker conformations may be consistent with the same experimental density [5, 62]. As demonstrated in prior large-scale correction efforts, such alternatives can lead to different local hydrogen-bonding patterns and functional interpretations [62]. In larger or more compact secondary-structure motifs, where local helical segments are embedded within constrained architectures and experimental structural coverage remains limited, such local ambiguities can propagate beyond individual residues by altering hydrogen-bonding patterns and influence the identification of biologically relevant conformations. Under such conditions, integrative modelling benefits from descriptors that are both physically interpretable and experimentally robust. In addition to pseudorotation parameters, backbone torsions such as δ and χ provide complementary structural information. The δ-torsion is directly coupled to sugar pucker geometry, while the χ-torsion reports on glycosidic orientation and base positioning, both of which are shown to differentiate between A- and B-like conformations (Fig. 6). Because χ-torsions, particularly for purines, are associated with large, electron-rich bases, they may be less ambiguous in experimental density than discrete pucker assignments. Overall, joint consideration of pseudotorsions, accessible pseudorotation states, puckering amplitude, and δ/χ-coupling provides a principled framework for discriminating among plausible structural solutions, particularly in regimes where low-amplitude puckers may facilitate A→B/B→A transitions.

### Comparison between the distribution of backbone torsions measured from X-ray and NMR structures

In order to represent the structural flexibility of solution behaviour in NMR-resolved structures, it is often recommended to submit an ensemble of models to the RCSB. The minimum number of models required (but not enforced) may depend on the nature of the study and the complexity of the system, typically around 20 for proteins [24]. From the curated structures in the NMR dataset (Supplementary Table S2), entries were most commonly deposited with only a single structure followed closely by ensembles of 10 structures. Overall, the range is quite large (between 1 and 28 structures) with ~10% having >20 (Supplementary Fig. S3). Standardizing the number of structures in each ensemble would facilitate comparative analysis, such as between the more rigid X-ray structures and the more flexible NMR structures, providing insight into the increased distribution of torsions due to flexibility. Ideally, each PDB code in the NMR dataset would have ensembles with the same number of structures to aid in the interpretation of the data, assuming all structures exhibit equal flexibility.

Given the lack of NMR structural data for Z-helices, this section primarily centers on the comparative analysis of backbone torsion distributions observed in X-ray and NMR structures for right-handed helices (A-helix and B-helix). However, scatterplot matrices for A-, B-, and Z-helices are also included for comparison (Supplementary Figs S4–S9).

For A-helices, there is a significant disparity between the number of X-ray and NMR structures (171 and 17, respectively). However, using the full ensemble of NMR models provides a more balanced comparison in terms of nucleotides (1849 for X-ray versus 2676 for NMR). In contrast, the B-helix grouping has a more even distribution of structures (135 for X-ray and 205 for NMR). When considering the ensemble of NMR models, the total increases to 1728, offering more insight into the distribution of backbone torsions. As it is known that B-helices are more flexible than A-helices [18, 19], the greater number of NMR models is beneficial for comparison. While a 1:1 distribution of data is not available, we cannot directly confirm the differences in flexibility between A- and B-helices; our findings align with the overall conclusions in the literature [19].

**Figure 10.**
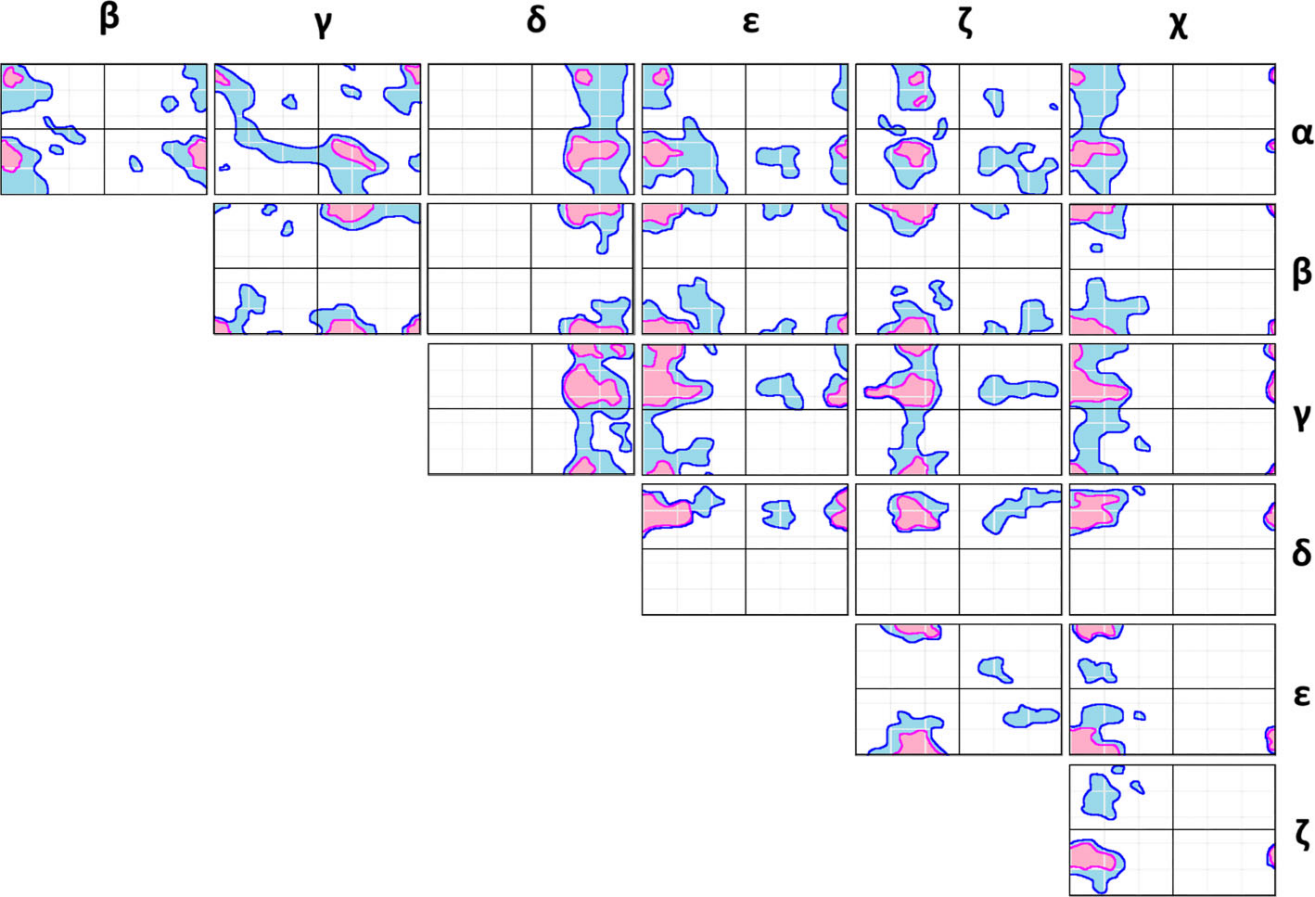
Traced contour of the distributions of A-DNA backbone torsions for X-ray structures (pink; inner contours) and NMR structures (blue; outer contours). Both *x*- and *y*-axis scale from −180° to +180°, with 0° indicated by black reference lines. An *x*-axis label for each column of plots is given on top, a *y*-axis label for each row of plots is given on the right.

**Figure 11.**
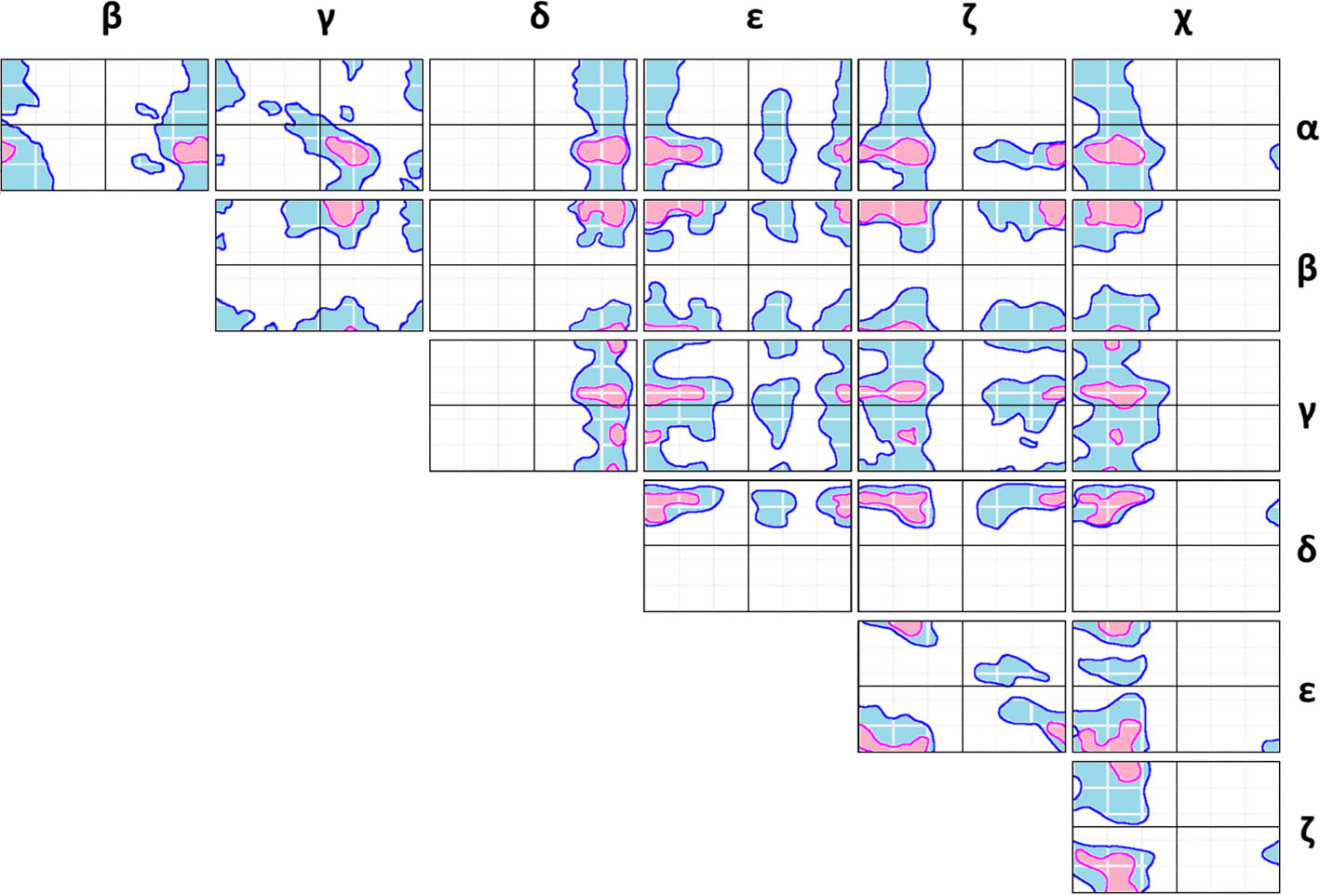
Traced contour of the distributions of B-DNA backbone torsions for X-ray structures (pink; inner contours) and NMR structures (blue; outer contours). Both *x*- and *y*-axis scale from −180° to +180°, with 0° indicated by black reference lines. An *x*-axis label for each column of plots is given on top, a *y*-axis label for each row of plots is given on the right.

As a proof of concept, a trace of the 2D plots (Figs 10 and 11) demonstrates the possible boundaries for allowed torsions for A- and B-helix type models using the ‘more rigid’ and ‘more relaxed’ distributions from X-ray and NMR datasets, respectively. Future work is required to determine whether all torsions, or only a subset, correspond to energetically strained conformations, analogous to Ramachandran outliers [6, 7, 63–65]. Such analysis should also account for intra- and intermolecular interactions in secondary and tertiary structures, rather than relying solely on steric criteria, as these interactions can offset unfavourable steric contacts [66].

### Comparison of A-helix backbone geometry

The dominant torsion peaks in the NMR dataset (Supplementary Fig. S10) are offset by ~7° relative to the circular mean values from the X-ray dataset (Fig. 3), with no consistent directional trend across torsions. The smallest deviations are the δ-torsion (∼0.5°) and the β-torsion (∼1°). The largest deviation is the χ-torsion (∼32°); however, considering the peak at −150°, this deviation is reduced to ∼9°. In addition, the peaks are significantly broader than those in the X-ray dataset, as visualized in Fig. 10, where the blue contour extends further out than the pink.

While the distributions obtained from the X-ray dataset appear normal (Fig. 3), the NMR dataset shows significant differences. Notably, their δ- and χ-torsions have broad unresolved peaks with two maxima. Additionally, there are sharp peaks within the distributions of the backbone torsions that were not expected and are not seen in the distribution of backbone torsions of B-helices. In particular, the ζ-torsion has two peaks that are almost equidistant (∼10°) from the X-ray circular mean torsion value (Supplementary Fig. S10).

The reported solution environments for the A-helix NMR structures were examined to provide experimental insight for the observed backbone torsion distributions (Supplementary Table S7). Across the dataset, these structures were obtained under broadly comparable solution conditions, typically involving aqueous buffers, moderate monovalent salt concentrations, mixed or fully deuterated solvent, and similar pH ranges. No single structure was determined under extreme conditions, such as unusually high salt or cosolvent-rich environments, that would be expected to uniquely bias backbone geometry. While reduced water activity is known to stabilize A-form DNA, the solution conditions represented here correspond to predominantly aqueous environments with moderate salt concentrations rather than strongly dehydrating regimes. Consequently, the broader torsional distributions observed for A-helix structures in solution cannot be attributed to a small number of experimental outliers. Instead, these results support the interpretation that the NMR ensemble reflects the intrinsic conformational flexibility of the A-helix in solution. While differences between crystal and solution environments may contribute to systematic shifts in mean values, the dominant effect within the NMR dataset appears to be ensemble broadening rather than condition-specific deformation.

Furthermore, in the NMR analysis, peaks are absent in regions associated with α-trans/γ-trans conformations, supporting the conclusion that, in the X-ray analysis, these likely arise from crystal contacts. Since NMR structures are inherently more flexible, such conformations would be expected to persist if they occurred independently of crystal packing. However, their absence may also reflect limitations of the force fields used in NMR structure refinement, which may not adequately sample or stabilize α-trans/γ-trans backbone conformations.

### Comparison of B-helix backbone geometry

The dominant torsion peaks in the NMR dataset (Supplementary Fig. S11) are offset by ~5° relative to the circular mean values from the X-ray dataset (Fig. 11), with no consistent directional trend across torsions. The smallest deviations are the δ- and ζ-torsions (∼3°). The largest deviation is the β-torsion (∼10°). In addition, the peaks are significantly broader than those in the X-ray dataset, as visualized in Fig. 11, where the blue contour extends further out than the pink.

The more distinguishable BI/BII peaks in β-, ε-, and ζ-torsions observed with the X-ray dataset are overshadowed by the broadening of the dominant peak, alterations in count, or a combination of both, making it challenging to accurately differentiate at the counting scale. By reducing the counting scale to 50 (Supplementary Fig. S12), the BI/BII peaks become somewhat more interpretable. However, a more rigorous statistical analysis of the backbone torsions would be required to properly ascertain the significance of the observed minor peaks and help with the sampling of common nucleic acid structures [67]. Notably, modelling the conformational space of B-DNA remains a challenge, and work on the CHARMM and AMBER force fields continues towards improving the sampling of BI/BII-DNA [59, 68, 69].

The solution behaviour of B-DNA lends credence to Bingman and co-workers’ speculation of the presence of the α-*trans*/γ-*trans* conformation [51]. This work is in therefore partial agreement, as the ratio of those peaks place it at <1%, which is much less than its almost 10% occurrence in A-helix crystal structures. Given B-DNA’s greater flexibility, one would expect a larger ratio of the α-trans/γ-trans conformation compared to A-helix. Additionally, since NMR structures are inherently more flexible, these conformations would be expected to be more prominent, but this is not the case. Again, they likely arise from crystal contacts and may be more common in datasets involving nucleic–nucleic, nucleic–protein, or nucleic–ligand interactions.

Interestingly, the ε-torsion has an ever-so-slight peak around 165° (Supplementary Fig. S12), which is reminiscent to that of C-DNA [19, 29, 33, 38]. Much like B-DNA, C-DNA exhibits BI- and BII-like conformers of C; however, the proportion of “BII” is >40% in C-DNA, compared to ~10% in B-DNA [70]. Unsurprisingly, despite having the increased conformational freedom, the δ-torsion consistently aligns with C2′-endo ring puckering (δ-torsion: ~145°; see ‘Analysis of common nucleic acid helix backbone torsions’ discussion) as expected. Similarly, the χ-torsion remains fairly consistent across conformers and is predominantly *anti*.

### Modelling challenges for RNA therapeutics and small molecule targeting

**Figure 12.**
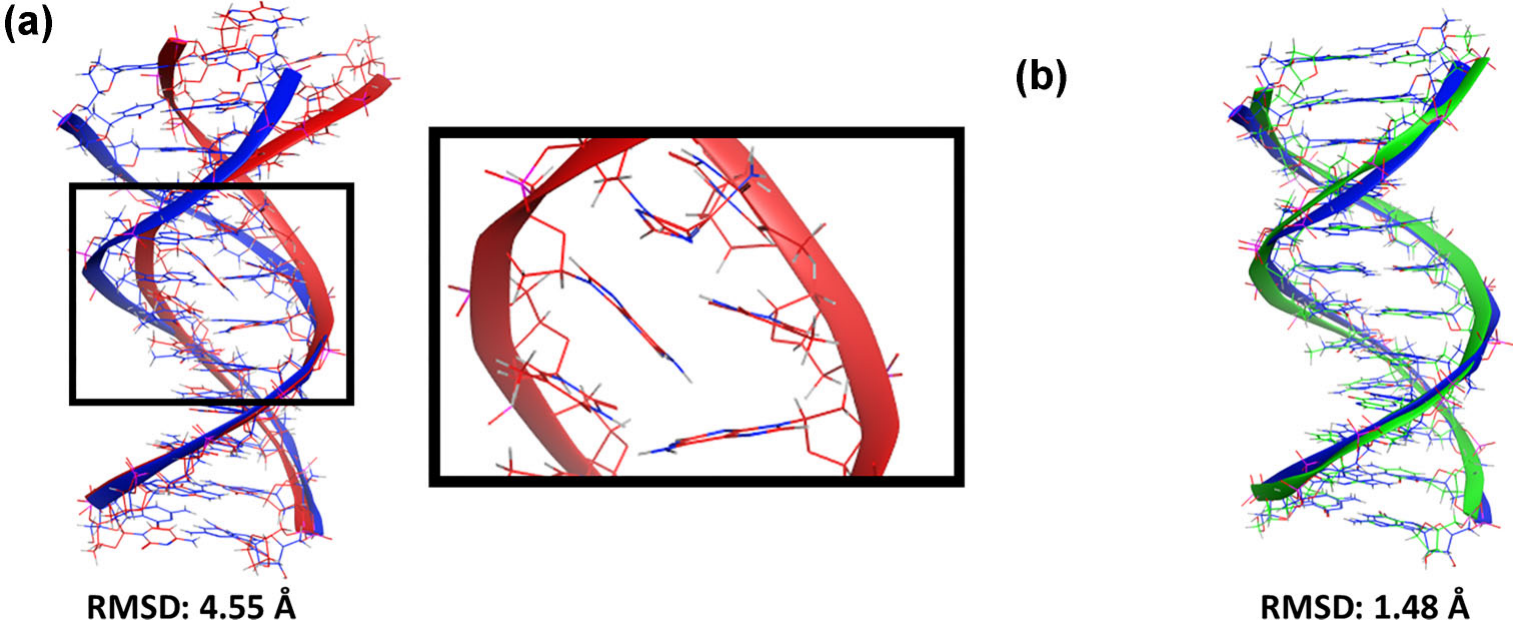
Modelling the Dickerson–Drew B-DNA dodecamer sequence (PDB ID: 1BNA [71]) using two different modelling approaches. (**a**) Modelling using only the backbone torsions (red) and (**b**) modelling using the backbone torsions with a base-step transformation (green), each superposed on the crystal structure (blue). DNA backbone atoms (P, O5′, C5′, C4′, C3′, O3′, C1′, C2′, and O4′) used for superposition/measuring backbone RMSD.

Improved accuracy in representing backbone torsions is a necessary step towards better nucleic acid models. However, refined torsional parameters alone do not guarantee the construction of physically realistic structures. Nucleic acid helices exhibit strong coupling between backbone geometry, base stacking, and base-pair positioning, such that small systematic errors in local geometry can accumulate and manifest as substantial global distortions. The complexity of biological folds arises from this interplay of coupled interactions and conformational dynamics. These limitations become readily apparent when considering a seemingly simple structure such as the Dickerson–Drew B-DNA dodecamer sequence (Fig. 12 and Supplementary Fig. S13) [71].

### Modelling only using the mean backbone torsions

A model of both complementary strands of the Dickerson–Drew B-DNA dodecamer [71] was generated by assigning the mean backbone torsions to each newly appended nucleotide at the 3′-end (build direction from the 5′-end to the 3′-end). The two chains were then superposed onto the crystal structure using the backbone atoms (P, O5′, C5′, C4′, C3′, O3′, C1′, C2′, and O4′) with a high backbone RMSD of 4.55 Å (Fig. 12a).

While the first set of modelled base pairs (C1:G24) have relatively small deviations, errors compound as the chain is extended, leading to a pronounced deviation from a linear B-DNA helical axis. This results in the modelled helix being 8.5 Å off-axis relative to the experimental structure and a drastically bent DNA helix conformation (Supplementary Fig. S14). DNA helices are known to bend, yet pieces shorter than 150–200 base pairs behave more like stiff rods that cannot be easily bent [72]. Smaller helical structures can bend in the presence of proteins without the need for disruption of the double-helical structure [19].

Furthermore, constraining the nucleobases to adopt only the mean χ-torsion does not permit canonical *cis* Watson–Crick/Watson–Crick (*cWW*) base pairing. To further help refine the model, a minimization step was implemented, focusing specifically on the nucleobases while keeping the backbone atoms tethered. Although this slightly improved the backbone RMSD, the orientation of the nucleobases still prevented *cWW* base pairing (Fig. 12a). The residual steric strain could not be relieved locally, underscoring that the observed distortions arise from the construction strategy rather than insufficient geometric relaxation.

### Modelling using the mean backbone torsions with a base-step transformation

The incorporation of affine transformations, such as translational vectors and rotational matrices, in addition to mean backbone torsions, mitigates the accumulation of geometric error observed earlier. In the context of MOE [46], this is achieved through the use of helix-type-specific affine transformations, which effectively act as base-step parameters [73] to ensure proper placement of the subsequent stacking base pairs. Using this approach, as implemented in the DNA/RNA Builder in MOE, a straight B-like helix is obtained with an accurate backbone RMSD of 1.48 Å relative to the experimental structure (Fig. 12b).

As in the previous case (Supplementary Fig. S13), a final minimization of nucleobases, with the backbone atoms tethered, reduces steric clashes and enhances the overall quality of the geometry, resulting in a more realistic and biologically relevant representation of the B-DNA structure. While the backbone torsional parameters are derived from ensemble statistics, the affine transformations were not. A translational vector and rotational matrix for both single-strand and double-stranded helices were determined from a single representative structure for each helix type from the w3DNA server [29, 33, 34]. This choice was deliberate due to the inherent complexity of averaging rotational matrices, which is non-trivial and requires careful consideration. Developing mean helix-type specific transformations in addition to mean backbone torsion values for building helix models (and other secondary structures), will require further thought and future work. To ensure that the applied affine transformations accurately capture the distinctive characteristics of each helix type, dinucleotides and dinucleotide pairs were selected from the middle of the polynucleotide chain for single-stranded and double-stranded helices, respectively. This choice aimed to minimize potential noise resulting from edge cases when compared to selecting dinucleotides from the termini. However, these considerations still enable the reproduction of the small bend in the helix axis of 1BNA [71] (verified using w3DNA server [29, 33]).

### A brief look into modelling RNA therapeutics and small molecule targeting RNA

The examples above illustrate how refined canonical helix parameterization is a necessary, but not sufficient, component of accurate nucleic acid modelling. When embedded with an appropriate geometric framework, these parameters define a structural baseline for detecting and interpreting deviations arising from chemical modification, sequence context, or ligand binding. This perspective aligns with recent efforts to formalize RNA structural descriptors for modelling and design, which emphasize the importance of well-defined reference states when comparing conformations across datasets and applications.

Recent work has highlighted how standardized geometric and topological descriptors can be used to quantify RNA conformational variability, facilitate comparison between modeled and experimental structures, and support rational design efforts in RNA targeting [74]. However, such frameworks implicitly assume that the underlying canonical conformations are themselves well characterized. The present analysis complements these approaches by establishing statistically robust backbone torsional distributions for canonical DNA and RNA helices and by demonstrating how deviations from these baselines manifest in practical modelling scenarios.

**Figure 13.**
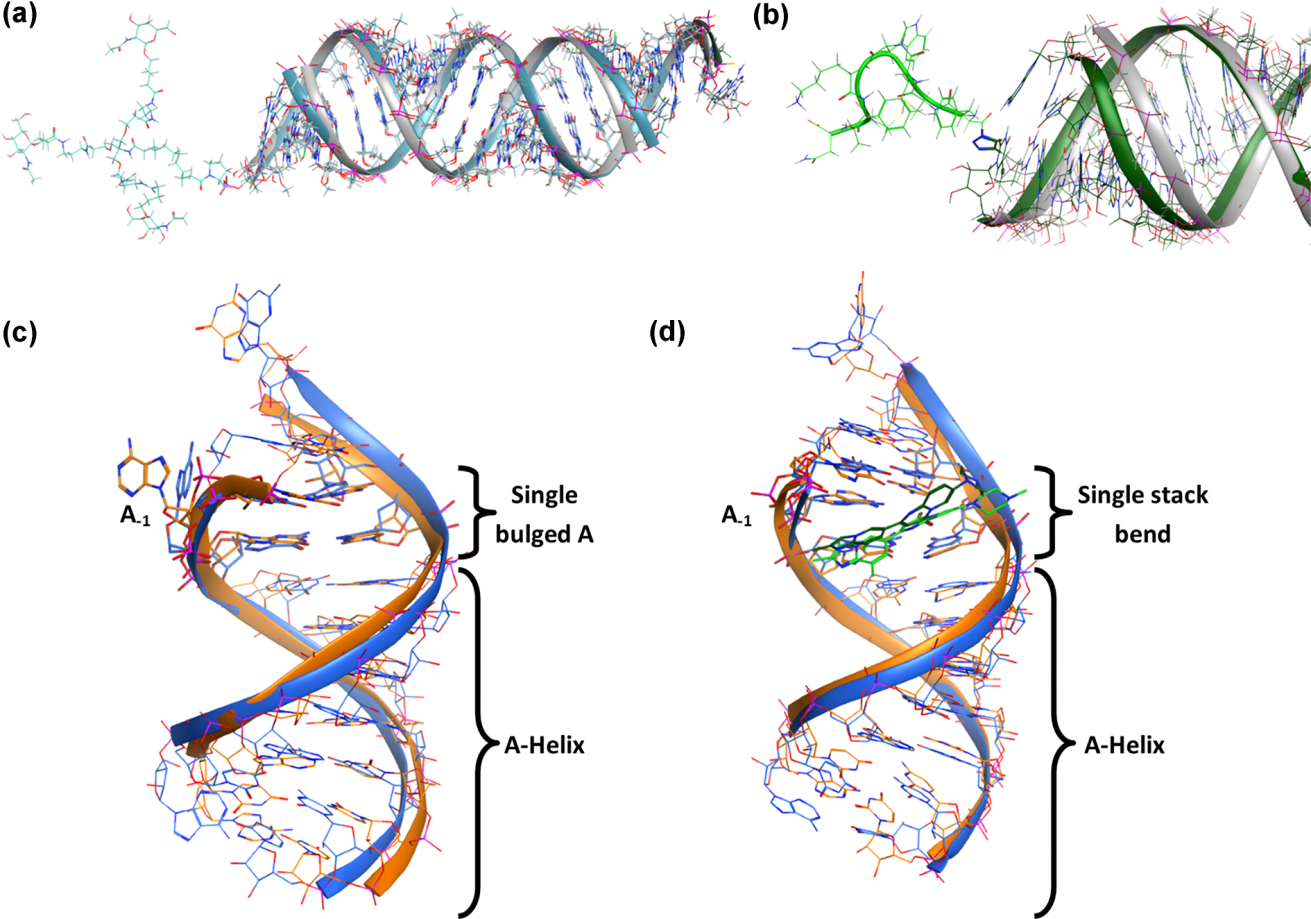
Select examples of RNA backbone distortions. (**a**) Modeled Inclisiran [75] (blue) superposed on modeled parent unmodified siRNA (grey); (**b**) modeled siRNA-peptide click conjugate [78] (green) on modeled parent unconjugated siRNA (grey); (**c**) modeled RNA structures (orange) superposed onto NMR structure of the U1 snRNA:E7 5′ SS complex (blue; PDB ID: 6HMI [79]); (**d**) modeled ligand-bound RNA structure (orange) superposed onto NMR structure of the U1 snRNA: E7 5′ SS complex (blue; PDB ID: 6HMO [79]). See Supplementary Materials for details of motif grafting and model construction for panels (c) and (d).

RNA therapeutics often face challenges in cellular uptake, prompting innovative approaches for enhanced efficacy. Strategies such as conjugation with a triantennary GalNAc (e.g. Inclisiran) [75, 76] or peptides aim to improve the cellular uptake of these therapeutic agents [27]. However, the introduction of conjugates may potentially influence the helix geometry of the polynucleic acid chains and their potency. On the other hand, with small molecules targeting RNA, deformations in the backbone can serve as the basis for selectivity (Fig. 13).

Modifications with phosphorothioate (PS), 2′-OMe, 2′-F and 2′-deoxy (DNA nucleotide), as employed in Inclisiran for the treatment of hypercholesterolemia [77], are designed to enhance stability and nuclease resistance while preserving the functional RNA duplex structure. As noted by Khvorova, the native helical structure has an essential role in potency [76]. Therefore, it is reasonable to expect that therapeutically relevant chemical modifications, when properly engineered, retain a geometry closely related to that of the parent unmodified siRNA.

Consistent with this expectation, modelling of both the parent siRNA and Inclisiran reveals minimal structural perturbations, with superposition of the duplex backbones having a backbone RMSD of 0.75 Å (Fig. 13a). This observation supports the use of a native-backbone conformational baseline for assessing the structural impact of chemical modification. Similarly, investigation of a siRNA-peptide click conjugate (Fig. 13b) reveals a backbone RMSD of 1.72 Å between the modelled unconjugated and peptide click-conjugated siRNA model. Terrazas *et al*. [78] make note that the incorporation of the c (RGDfK) peptide to the 5′-end of the siRNA does not adversely affect duplex stability, a conclusion that is consistent with the preserved helical geometry observed here.

With a curated library of canonical helix parameters, reference models provide a quantitative baseline that enables both the identification and reconstruction of localized backbone distortions in biologically relevant RNA structures, thereby facilitating accurate measurement of features such as bulges arising from disease-causing mutations [79, 80–82].

In the unbound state, the U1 snRNA:E7 5′-splice site (5′-SS) interface (PDB ID: 6HMI [79]) has a single bulged A (Fig. 13c; labelled A_-1_) that lies outside the allowed torsional ranges and is flagged by MolProbity. While such validation tools identify outliers, they do not quantify the extent or structural origin of the deviation, nor do they provide a consistent reference for interpreting coupled changes across multiple backbone torsions. The measured backbone torsions deviate by 2.6°–43.3° (average 17.9°) from the A-helix circular means derived from the high-resolution X-ray dataset. In particular, the large deviation of the δ-torsion (124.6° versus 81.6°) indicates a shift to C1′-exo sugar puckering, while differences in the α-, γ-, and ζ-torsions reflect a locally twisted backbone at the bulged nucleotide.

In contrast, the bound state (Fig. 13d; PDB ID: 6HMO [79]) adopts a single stack-bend conformation that more closely resembles canonical A-helix geometry. The corresponding model reproduces the experimental structure with an overall backbone RMSD below 3 Å, with improved agreement relative to the unbound form, following motif-based reconstruction onto a canonical A-helix framework.

These examples demonstrate that deviations from canonical torsional parameters provide a direct and interpretable measure of localized structural variation, capturing both the preservation of native-like geometry in chemically modified systems and the identification of coordinated, multi-torsional distortions associated with bulging and ligand binding. Such complementary behaviours establish a consistent reference for distinguishing genuine structural adaptations from model artifacts. In addition, these observations further suggest an alternative perspective for nucleic acid modelling, in which nucleobase placement and base–base interactions define the primary structural constraints, and the backbone is subsequently accommodated to satisfy these interactions. In this view, canonical torsional parameters serve not as generative inputs, but as a reference framework for evaluating and refining backbone geometry.

## Conclusion

This work provides valuable insights into the differences in nucleic acid helix backbone torsions, elucidating their variability, the factors influencing them, and the difficulties associated with precise modelling. The analysis of nucleic acid backbone torsions was performed across a vast dataset comprising 9923 structures and 4 184 581 nt. This revealed the consistency of backbone torsion angles despite the diverse set of secondary structures, particularly in single-stranded RNA.

A curated dataset of A-, B-, and Z-helix structures from X-ray diffraction and NMR solution methods was employed to support the reliability of previously reported mean torsion values. Additionally, updated circular mean torsion values were provided for the three helix structures for natural nucleotides in the absence of proteins and small-molecule ligands. An examination of crystal systems containing notable variations in A-helix geometry (α-trans/γ-trans) supported earlier reports that these variations arise from crystal packing but clarified that they do not exclusively occur in orthorhombic crystals.

The analysis of B-helix backbone geometry highlighted the flexibility of B-form compared to A-form. The comparison between X-ray and NMR structures for A- and B-helices revealed significant differences in torsion distributions, emphasizing the challenges in accurately representing the conformational space of B-helices. A brief exploration of Z-helix backbone geometry underscored the distinctive torsion values of the two major conformers (ZI and ZII-DNA).

The examination of modelling challenges for RNA therapeutics and small-molecule targeting emphasized the importance of accurate backbone torsion representation. The study reaffirmed that relying solely on mean backbone torsions for modelling would result in unreliable structures. More broadly, the updated torsional distributions reported here provide an experimentally derived reference framework for distinguishing canonical conformations from structural adaptations associated with crystal packing, conformational flexibility, chemical modification, and molecular recognition.

## Abbreviations

A, Adenosine; A&H, Arnott and Hukins; ASO, Antisense Oligonucleotides; C, Cytidine; cWW, *cis* Watson–Crick:Watson-Crick; DA, Deoxyadenosine; DC, Deoxycytidine; DG, Deoxyguanosine; DT, Deoxythymine; G, Guanosine; GalNAc, N-acetylgalactosamine; miRNA, micro RNA; MOE, Molecular Operating Environment; NDB, Nucleic acid Data Bank; NOE, Nuclear Overhauser Effect; PDB, Protein Data Bank; R, Purine; RCSB, Research Collaboratory for Structural Bioinformatics; RMSD, Root Mean Square Deviation; siRNA, small interfering RNA; snRNA, small nuclear RNA; SMARTS, SMILES Arbitrary Target Specification; SMILES, Simplified Molecular-Input Line-Entry System; U, Uridine; w3DNA, web 3DNA; Y, Pyrimidine.

## Supplementary Material

gkag668_Supplemental_Files

## Data Availability

The curated X-ray and NMR datasets generated and analysed in this study are available as Supporting Data accompanying this article. These datasets contain nucleic acid backbone torsion angles, sugar pucker parameters, helix classifications, and associated metadata derived from NDB/PDB structures, together with a detailed README file describing file formats and fields. The complete dataset, comprising 4 184 581 entries, is available through Zenodo (DOI: 10.5281/zenodo.20469706) in compressed format (data_FULL.csv.xz). Data presented in the main text and Supporting Materials were derived from these deposited datasets.
