# Supplementary material for: Conformational analysis of nucleic acids for optimizing DNA and RNA topological models
Source: Nucleic Acids Res. 2026 Jul 14;54(13):gkag668. doi: 10.1093/nar/gkag668 (PMC13365949; doi:10.1093/nar/gkag668)
Supplement: gkag668_Supplemental_Files [file gkag668_supplemental_files.zip › NAR-01888-L-2026_Supplemental_Materials_20260621.pdf]

Supplementary Materials

Conformational analysis of nucleic acids for optimizing DNA and RNA topological models

Philippe Archambault[*1,2], *Matthias Keil*[1], *Heidi M. Muchall*[2] *and Gilles H. Peslherbe*[2]

[1] Chemical Computing Group, 910-1010 Sherbrooke W., Montréal, Québec, Canada H3A 2R7.

[2] Centre for Research in Molecular Modeling and Department of Chemistry and Biochemistry, Concordia University, 7141 Sherbrooke West, Montréal, Québec, Canada H4B 1R6.

* Author to whom correspondence should be addressed. E-mail: parchambault@chemcomp.com

Table of Contents

1. PDB codes for X-ray and NMR Datasets **6**

- **Supplementary Table S1**: List of PDB codes in X-ray dataset.
- **Supplementary Table S2**: List of PDB codes in NMR dataset.

2. Nucleotide SMARTS patterns **8**

- **Supplementary Table S3**: DNA SMARTS patterns.
- **Supplementary Table S4**: RNA SMARTS patterns.
- **Supplementary Table S5**: RNA (non-Oxygen for O2') SMARTS patterns.

3. Field histograms of full dataset **10**

- **Supplementary Figure S1**: Field histogram of 4,184,581 nucleotide (9,923 nucleic acid structures) torsion angles (°). The x-axis is shared for each torsion and ranges from -180° to 180° and the y-axis is the count of the frequency of occurrence by torsion angle (scale: 0 to 400,000).

4. Crystal systems with α-trans/γ-trans conformations **11**

- **Supplementary Figure S2**: Spatial relationship between α-trans/γ-trans torsions and crystal packing contacts in A-DNA. (a) Full A-DNA duplex (PDB code: 4OKL) highlighting residues adopting α-trans/γ-trans torsions (gold) and neighbouring crystal contacts (green) within 4.5 Å of the asymmetric unit. The grey mesh represents the molecular surface generated from atoms belonging to crystal contact atoms within 3.0 Å of the asymmetric unit (regions of close crystal packing). (b) Zoomed view of a representative residue exhibiting α-trans/γ-trans torsions (gold) and its local crystal environment (green).
- **Supplementary Table S6**: Crystal systems of selected A-helix structures.

5. Distribution of NMR models per PDB entry in NMR dataset **13**

- **Supplementary Figure S3**: Distribution of the number of models per PDB code (ensemble of NMR determined structures) in the NMR dataset for A-helix (red), B-helix (blue) and Z-helix (green).

6. Scatter matrices of nucleic acid torsions **14**

- **Supplementary Figure S4**: Scatter matrix of nucleic acid torsions of A-helices from X-ray diffraction methods (resolution: 2 Å or less) obtained from the RCSB.[2–4] Both x- and y-axis scale from -180° to +180°, with 0° indicated by black reference lines. An x-axis label for each column of plots is given on top, a y-axis label for each row of plots is given on the right.
- **Supplementary Figure S5**: Scatter matrix of nucleic acid torsions of A-helices from NMR solution methods obtained from the RCSB.[2–4] Both x- and y-axis scale from -180° to +180°, with 0° indicated by black reference lines. An x-axis label for each column of plots is given on top, a y-axis label for each row of plots is given on the right.
- **Supplementary Figure S6**: Scatter matrix of nucleic acid torsions of B-helices from X-ray diffraction methods (resolution: 2 Å or less) obtained from the RCSB.[2–4] Both x- and y-axis scale from -180° to +180°, with 0° indicated by black reference lines. An x-axis label for each column of plots is given on top, a y-axis label for each row of plots is given on the right.
- **Supplementary Figure S7**: Scatter matrix of nucleic acid torsions of B-helices from NMR solution methods obtained from the RCSB.[2–4] Both x- and y-axis scale from -180° to +180°, with 0° indicated by black reference lines. An x-axis label for each column of plots is given on top, a y-axis label for each row of plots is given on the right.
- **Supplementary Figure S8**: Scatter matrix of nucleic acid torsions of Z-helices (separated into purine and pyrimidine; green and dark green, respectively) from X-ray diffraction methods (resolution: 2 Å or less) obtained from the RCSB.[2–4] Both x- and y-axis scale from -180° to +180°, with 0° indicated by black reference lines. An x-axis label for each column of plots is given on top, a y-axis label for each row of plots is given on the right.

- **Supplementary Figure S9**: Scatter matrix of nucleic acid torsions of Z-helices (separated into purine and pyrimidine; green and dark green, respectively) from NMR solution methods obtained from the RCSB.[2–4] Both x- and y-axis scale from -180° to +180°, with 0° indicated by black reference lines. An x-axis label for each column of plots is given on top, a y-axis label for each row of plots is given on the right.

7. Field histograms A- and B-Helix (NMR dataset) 20

- **Supplementary Figure S10**: Field histogram of NMR A-helix torsion angles (°). The x-axis is shared for each torsion and ranges from -180° to 180° and the y-axis is the count of the frequency of occurrence by torsion angle (scale: 0 to 200). The solid vertical line represents the angle peak for each torsion measured in this work from the X-ray A-helix structures. Regions associated with α-trans/γ-trans conformation as well as ζ-torsion peaks are identified.
- **Supplementary Table S7:** NMR A-form structures and reported experimental conditions
- **Supplementary Figure S11**: Field histogram of NMR B-helix torsion angles (°). The x-axis is shared for each torsion and ranges from -180° to 180° and the y-axis is the count of the frequency of occurrence by torsion angle (scale: 0 to 2,000). The solid vertical line represents the angle peak for each torsion measured in this work from the X-ray B-helix structures.
- **Supplementary Figure S12**: Field histogram of NMR B-helix torsion angles (°). The x-axis is shared for each torsion and ranges from -180° to 180° and the y-axis is the count of the frequency of occurrence by torsion angle (scale: 0 to 50). The solid vertical line represents the angle peak for each torsion measured in this work from the X-ray B-helix structures. Peaks associated with BI-/BII-DNA as well as regions associated with α-trans/γ-trans conformations are identified.

8. Modeled 1BNA **24**

- **Supplementary Figure S13**: Modeling the Dickerson-Drew B-DNA dodecamer sequence (PDB ID: 1BNA[1]) using two different modeling approaches. The crystal structure (1BNA) is shown in blue and the modeled helices are shown in red, orange and green which have the worst to best levels of accuracy, respectively. Pathway 1 approach (above) uses only the backbone torsions. Pathway 2 approach (below) uses the backbone torsions with a base-step transformation. DNA backbone atoms (P, O5', C5', C4', C3', O3', C1', C2', and O4') used for superposition/measuring backbone RMSD.
- **Supplementary Figure S14**: Deviation of helical axis in modeled Dickerson-Drew B-DNA dodecamer sequence (PDB ID: 1BNA[5]) using two different modeling approaches. (a) Superposition (DNA backbone atoms) of X-ray (blue), pathway 1 modeling (red) and pathway 2 modeling (green) structures (see Supplementary Figure S13 for pathway descriptions). (b) Rendering of base-pair origin[6] for each structure to highlight the axis displacement of each model with respect to the X-ray structure. Distance shown in Å. DNA backbone atoms (P, O5', C5', C4', C3', O3', C1', C2', and O4') used for superposition/measuring backbone RMSD.

9. Motif Grafting and Model Construction Protocol **26**

10. Additional references **28**

1. PDB codes for X-ray and NMR Datasets

Supplementary Table S1: List of PDB codes in X-ray dataset.

**A-helix**									
115D	118D	137D	138D	160D	1D78	1D79	1D90	1D9H	1DNZ
1I0F	1I0G	1I0J	1I0K	1I0M	1I0N	1I0P	1I0Q	1I5W	1ICG
1ID9	1IDW	1IH4	1IH6	1IHA	1M77	1MA8	1MLX	1NZG	1R3G
1R3Z	1VJ4	1VT8	1XJX	1XUW	1XUX	1Y7F	1Y86	1Y8V	1Y9F
1Y9S	1YBC	1ZEX	1ZEY	1ZF1	1ZF6	1ZF8	1ZF9	1ZFA	212D
213D	221D	222D	232D	243D	248D	254D	260D	295D	2A7E
2AXB	2B1B	2D94	2DLJ	2FIJ	2FIL	2GPX	2H05	2HC7	2NSK
2PKV	2PL4	2PLO	317D	327D	332D	337D	338D	340D	341D
344D	345D	348D	349D	368D	369D	370D	371D	383D	395D
396D	399D	3BM0	3FA1	3HG8	3HGD	3HR3	3IFF	3IFI	3IJK
3IJN	3IKI	3K18	3KQ8	3LTR	3LTU	3OT0	3OZ4	3Q61	3SD8
3SSF	3UKB	3UKC	3UKE	3V06	3V07	411D	412D	414D	440D
441D	4F2Y	4F4N	4F8G	4HQH	4I1G	4OKL	4QKK	4RHD	4U6K
4U6L	4U6M	4YS5	5AXE	5AXF	5DEK	5FHL	5IYE	5IYG	5IYJ
5MVK	5MVL	5MVP	5MVQ	5MVT	5U0Q	5WSP	5WSS	5WV7	5XK0
5XK1	6D54	6DWT	6DXJ	6IBQ	6L75	6ZPF	6ZQ9	6ZR1	6ZRL
6ZRS	6ZW3	6ZWU	6ZX5	6ZX8	7A9L	7A9N	7A9O	7A9P	7A9T
9DNA									
**B-helix**									
122D	123D	158D	183D	196D	1BNA	1CW9	1D23	1D3R	1D49
1D56	1D61	1D8G	1D8X	1D9R	1DOU	1DPN	1EHV	1EN3	1EN9
1ENN	1G75	1G8N	1G8U	1G8V	1I3T	1IKK	1J8L	1N1O	1N5C
1NQS	1NVN	1NVY	1P4Y	1P54	1S1K	1S23	1SGS	1SK5	1VE8
1ZF0	1ZF2	1ZF3	1ZF4	1ZF5	1ZF7	1ZFB	1ZFF	1ZFG	251D
2D25	2FIH	2FII	2OKS	2ORF	2ORH	2P8D	2QEF	2QEG	307D
388D	389D	3BSE	3DNB	3FL6	3GGK	3GJL	3I5E	3IGT	3N4N
3OPI	3PBX	3TOK	3U2N	3U89	423D	431D	436D	456D	460D
461D	463D	476D	477D	4C5X	4C63	4C64	4F2X	4F3U	4GJU
4GLC	4GLG	4GLH	4H5A	4HLI	4KW0	4KWX	4MGW	4MKW	4O5Y
4O5Z	4PWM	4QC7	4R49	4R4D	5CH0	5CJY	5DNB	5DSA	5DSB
5ET9	5EWB	5EZF	5FHJ	5VBJ	5W1Z	5W20	5XUV	5ZAT	6ADV
6CQ3	6JV5	6QJS	6QT1	6QT2	6QT3	6QT4	6QT5	6QT6	6X5D
7BNA	7JLH	7KCI	7RQT	9BNA					
**Z-helix**									
133D	145D	181D	192D	1D24	1D39	1D41	1D48	1D76	1DA2
1DCG	1DN4	1DN5	1DNF	1JES	1LJX	1M6R	1OMK	1VTT	1VTV
1VTW	1XA2	223D	242D	2HTO	2OBZ	312D	313D	314D	331D
351D	362D	3F8O	3FQB	3GCY	3GDA	3QBA	3WBO	400D	417D
4DKZ	4DWY	4E2R	4E4O	4FS5	4FS6	4OCB	4R15	4XSN	5JZQ
6AQT	6AQV	6AQW	6AQX	7JY2					

Supplementary Table S2: List of PDB codes in NMR dataset.

**A-helix**									
124D	140D	142D	1D68	1DHH	1L3M	2LAR	2LB4	2M1V	2M84
2M8A	2O7W	5B81	5TGG	6GMY	7NBL	7NEJ			
**B-helix**									
103D	132D	141D	169D	170D	171D	175D	176D	1D18	1D19
1D20	1D69	1DRN	1DUF	1K8J	1K8L	1K8N	1KBD	1KXS	1LAI
1LAQ	1LAS	1LWA	1N14	1N17	1N1K	1N2W	1NAJ	1NEV	1NGO
1ONM	1OPQ	1OQ2	1OSR	1PYJ	1QL5	1RN9	1RVH	1RVI	1S0T
1S37	1S74	1S75	1S88	1S9O	1SP6	1SS7	1SSJ	1SSV	1SY8
1TQR	1TUQ	1U6N	1U6O	1UAB	1X2O	1X2S	1X2U	1X2V	1X2X
1X2Y	1X2Z	1X30	1XCI	1YCT	1YCW	1ZYF	1ZYG	1ZYH	2HKB
2HLI	2HMD	2HMR	2HOU	2HPX	2HSK	2HSL	2HSR	2HSS	2ICZ
2JYK	2K0V	2K1Y	2KAL	2KBD	2KD9	2KDA	2KH3	2KH5	2KH6
2KH7	2KH8	2KUZ	2KV0	2L2U	2L2V	2L7D	2L8I	2L8P	2L8Q
2LFA	2LFX	2LFY	2LG0	2LHO	2LIA	2LIB	2LL9	2LSC	2LSF
2LSZ	2LT0	2LWG	2LWM	2LWN	2LWO	2M11	2M2C	2M3P	2M3Y
2M40	2M43	2M44	2M54	2MAV	2MCI	2MCJ	2MH6	2MHX	2MHZ
2MJX	2MMF	2MMQ	2MMR	2MMS	2MNB	2MNF	2MNX	2MO2	2MO7
2N0Q	2N4M	2N5O	2N5P	2N9F	2N9H	2NQ1	2NQ4	2O4Y	2RRQ
2RRR	2RT8	2RVP	3KBD	4KBD	5HQF	5HQQ	5IV1	5IZP	5J3F
5J3G	5J3I	5KGV	5KI4	5KI5	5KI7	5KIB	5KIE	5KIF	5KIH
5L06	5L2G	5TRN	5UZ1	5UZ2	5UZ3	5UZD	5UZF	5UZI	5ZLD
6ALS	6ALT	6ALU	6ASF	6DM7	6ED9	6FY6	6FY7	6I4O	6RLS
6XAH	7B4Z	7B71	7B72	7BFS	7CUK	7KWL	7KWR	7NBP	7OGV
7OHE	7OHJ	7OHM	7QA9	7W0V					
**Z-helix**									
1R4D									

2. Nucleotide SMARTS patterns

Supplementary Table S3: DNA SMARTS patterns.

Polymer type	SMARTS patterns
**Polymer**	[PX4]([#G6Q1])([#G6Q1])([OQ2])-[OQ2]-[CX4]-[CX4;q2r5]1-@[CX4;q2r5]([OQ2!i])-@[CX4]-@[CX4]-@[OQ2!i]1
**Free**	[OQ1]-[CX4]-[CX4;q2r5]1-@[CX4;q2r5]([OQ1!i])-@[CX4](-[OQ1,OQ2])-@[CX4]-@[OQ2!i]1
**5'end**	[OQ1]-[CX4]-[CX4;q2r5]1-@[CX4;q2r5]([OQ2!i])-@[CX4]-@[CX4]-@[OQ2!i]1<br><br>[CX4](-[OQ2])-[CX4;q2r5]1-@[CX4;q2r5]([OQ2!i])-@[CX4]-@[CX4]-@[OQ2!i]1
**3'end**	[PX4]([#G6Q1])([#G6Q1])([OQ2])-[OQ2]-[CX4]-[CX4;q2r5]1-@[CX4;q2r5]([OQ1!i])-@[CX4]-@[CX4]-@[OQ2!i]1
**Free+PO_4**	[PX4]([#G6Q1])([#G6Q1])([#G6Q1])-[OQ2]-[CX4]-[CX4;q2r5]1-@[CX4;q2r5]([OQ1!i])-@[CX4]-@[CX4]-@[OQ2!i]1<br><br>[PX4h,H]([#G6Q1])([#G6Q1])-[OQ2]-[CX4]-[CX4;q2r5]1-@[CX4;q2r5]([OQ1!i])-@[CX4]-@[CX4]-@[OQ2!i]1
**5'+PO_4**	[PX4]([#G6Q1])([#G6Q1])([#G6Q1])-[OQ2]-[CX4]-[CX4;q2r5]1-@[CX4;q2r5]([OQ2!i])-@[CX4]-@[CX4]-@[OQ2!i]1<br><br>[PX4h,H]([#G6Q1])([#G6Q1])-[OQ2]-[CX4]-[CX4;q2r5]1-@[CX4;q2r5]([OQ2!i])-@[CX4]-@[CX4]-@[OQ2!i]1

Supplementary Table S4: RNA SMARTS patterns.

Polymer type	SMARTS patterns
**Polymer**	[PX4]([#G6Q1])([#G6Q1])([OQ2])-[OQ2]-[CX4]-[CX4;q2r5]1-@[CX4;q2r5]([OQ2!i])-@[CX4](-[OQ1,OQ2])-@[CX4]-@[OQ2!i]1
**Free**	[OQ1]-[CX4]-[CX4;q2r5]1-@[CX4;q2r5]([OQ1!i])-@[CX4](-[OQ1,OQ2])-@[CX4]-@[OQ2!i]1
**5'end**	[OQ1]-[CX4]-[CX4;q2r5]1-@[CX4;q2r5]([OQ2!i])-@[CX4](-[OQ1,OQ2])-@[CX4]-@[OQ2!i]1<br><br>[CX4](-[OQ2])-[CX4;q2r5]1-@[CX4;q2r5]([OQ2!i])-@[CX4](-[OQ1,OQ2])-@[CX4]-@[OQ2!i]1
**3'end**	[PX4]([#G6Q1])([#G6Q1])([OQ2])-[OQ2]-[CX4]-[CX4;q2r5]1-@[CX4;q2r5]([OQ1!i])-@[CX4](-[OQ1,OQ2])-@[CX4]-@[OQ2!i]1
**Free+PO_4**	[PX4]([#G6Q1])([#G6Q1])([#G6Q1])-[OQ2]-[CX4]-[CX4;q2r5]1-@[CX4;q2r5]([OQ1!i])-@[CX4](-[OQ1,OQ2])-@[CX4]-@[OQ2!i]1<br><br>[PX4h,H]([#G6Q1])([#G6Q1])-[OQ2]-[CX4]-[CX4;q2r5]1-@[CX4;q2r5]([OQ1!i])-@[CX4](-[OQ1,OQ2])-@[CX4]-@[OQ2!i]1
**5'+PO_4**	[PX4]([#G6Q1])([#G6Q1])([#G6Q1])-[OQ2]-[CX4]-[CX4;q2r5]1-@[CX4;q2r5]([OQ2!i])-@[CX4](-[OQ1,OQ2])-@[CX4]-@[OQ2!i]1<br><br>[PX4h,H]([#G6Q1])([#G6Q1])-[OQ2]-[CX4]-[CX4;q2r5]1-@[CX4;q2r5]([OQ2!i])-@[CX4](-[OQ1,OQ2])-@[CX4]-@[OQ2!i]1

Supplementary Table S5: RNA (non-Oxygen for O2') SMARTS patterns.

Polymer type	SMARTS patterns
**Polymer**	[PX4]([#G6Q1])([#G6Q1])([OQ2])-[OQ2]-[CX4]-[CX4;q2r5]1-@[CX4;q2r5]([OQ2!i])-@[CX4](-[#X])-@[CX4]-@[OQ2!i]1
**Free**	[OQ1]-[CX4]-[CX4;q2r5]1-@[CX4;q2r5]([OQ1!i])-@[CX4](-[#X])-@[CX4]-@[OQ2!i]1
**5'end**	[OQ1]-[CX4]-[CX4;q2r5]1-@[CX4;q2r5]([OQ2!i])-@[CX4](-[OQ1,OQ2])-@[CX4]-@[OQ2!i]1<br><br>[CX4](-[OQ2])-[CX4;q2r5]1-@[CX4;q2r5]([OQ2!i])-@[CX4](-[#X])-@[CX4]-@[OQ2!i]1
**3'end**	[PX4]([#G6Q1])([#G6Q1])([OQ2])-[OQ2]-[CX4]-[CX4;q2r5]1-@[CX4;q2r5]([OQ1!i])-@[CX4](-[#X])-@[CX4]-@[OQ2!i]1
**Free+PO_4**	[PX4]([#G6Q1])([#G6Q1])([#G6Q1])-[OQ2]-[CX4]-[CX4;q2r5]1-@[CX4;q2r5]([OQ1!i])-@[CX4](-[#X])-@[CX4]-@[OQ2!i]1<br><br>[PX4h,H]([#G6Q1])([#G6Q1])-[OQ2]-[CX4]-[CX4;q2r5]1-@[CX4;q2r5]([OQ1!i])-@[CX4](-[#X])-@[CX4]-@[OQ2!i]1
**5'+PO_4**	[PX4]([#G6Q1])([#G6Q1])([#G6Q1])-[OQ2]-[CX4]-[CX4;q2r5]1-@[CX4;q2r5]([OQ2!i])-@[CX4](-[#X])-@[CX4]-@[OQ2!i]1<br><br>[PX4h,H]([#G6Q1])([#G6Q1])-[OQ2]-[CX4]-[CX4;q2r5]1-@[CX4;q2r5]([OQ2!i])-@[CX4](-[#X])-@[CX4]-@[OQ2!i]1

3. Field histograms of full dataset.

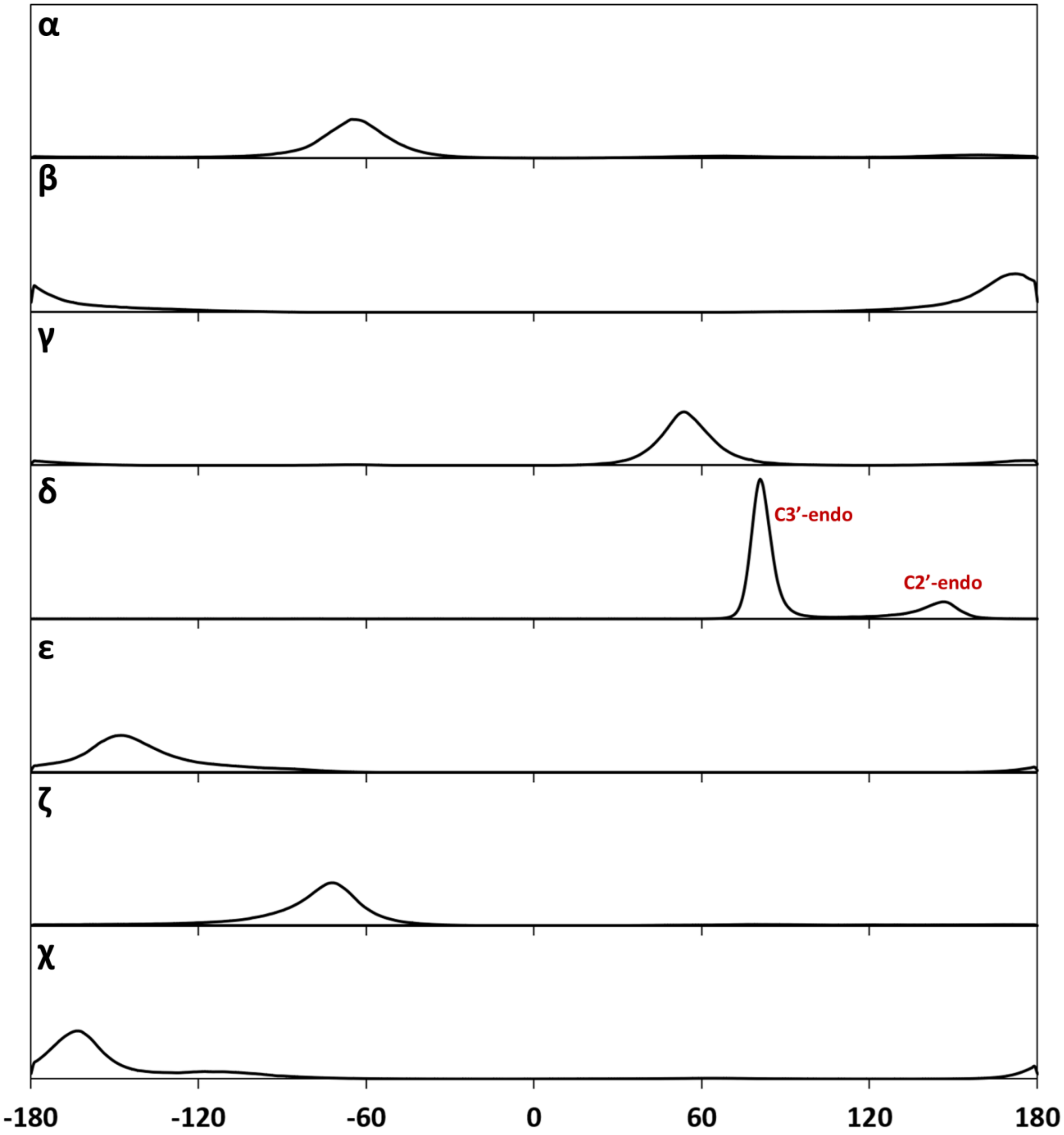

Supplementary Figure S1: Field histogram of 4,184,581 nucleotide (9,923 nucleic acid structures) torsion angles (°). The x-axis is shared for each torsion and ranges from -180° to 180° and the y-axis is the count of the frequency of occurrence by torsion angle (scale: 0 to 400,000).

4. Crystal systems with α-trans/γ-trans conformations

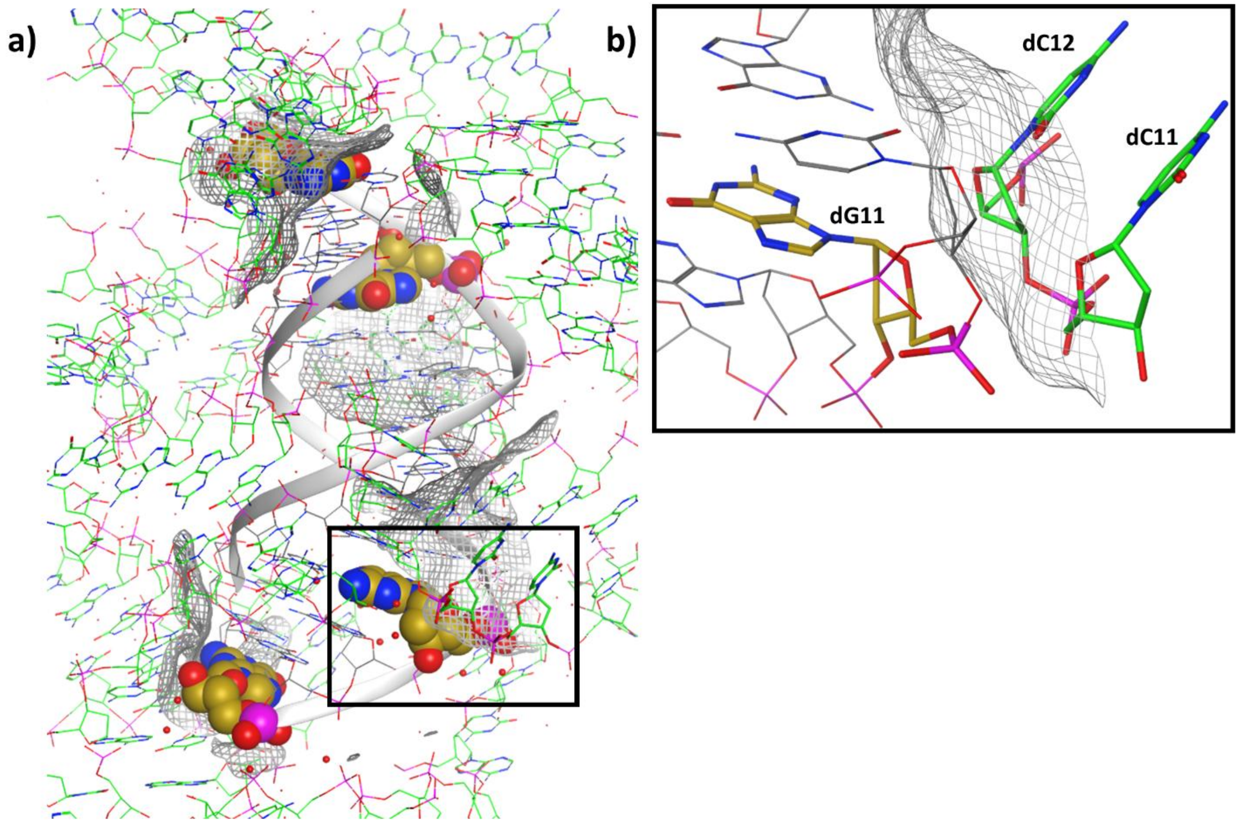

Supplementary Figure S2: Spatial relationship between α-trans/γ-trans torsions and crystal packing contacts in A-DNA. (a) Full A-DNA duplex (PDB code: 4OKL) highlighting residues adopting α-trans/γ-trans torsions (gold) and neighbouring crystal contacts (green) within 4.5 Å of the asymmetric unit. The grey mesh represents the molecular surface generated from atoms belonging to crystal contact within 3.0 Å of the asymmetric unit (regions of close crystal packing). (b) Zoomed view of a representative residue exhibiting α-trans/γ-trans torsions (gold) and its local crystal environment (green).

Supplementary Table S6: Crystal systems of selected A-helix structures.[a]

Number	Space Group[b]	Crystal System	PDB codes									
4	$P\ 1\ 2_1\ 1$	monoclinic	1XUX	414D	5U0Q							
5	$C\ 1\ 2\ 1$		1ICG	1ID9	1IDW	1IHA						
19	$P\ 2_1\ 2_1\ 2_1$	orthorhombic	137D	160D	1D9H	1I0F	1I0G	1I0J	1I0K	1I0M	1I0N	1I0P
			1I0Q	1I5W	1MA8	1MLX	1NZG	1R3G	1XUW	1Y7F	1Y86	1Y8V
			1Y9F	1Y9S	1YBC	1ZEX	1ZEY	1ZF1	1ZF8	1ZF9	213D	221D
			222D	2AXB	368D	369D	370D	371D	383D	399D	3OT0	3OZ4
			3Q61	3SD8	3UKB	3UKC	3UKE	3V06	3V07	411D	412D	440D
			4F2Y	4HQH	5AXE	5AXF						
20	$C\ 2\ 2\ 2_1$		254D									
76	$P\ 4_1$	tetragonal	5IYE									
78	$P\ 4_3$		1XJX									
96	$P\ 4_3\ 2_1\ 2$		4OKL	5IYG	5IYJ	5WV7	118D	1M77	1R3Z	243D	295D	2A7E
			2D94	2DLJ	2GPX	2H05	2HC7	2NSK	317D	337D	338D	340D
			341D	344D	345D	3BM0	3FA1	3HG8	3IFI	3IJK	3IJN	3IKI
			3K18	3KQ8	3LTR	3LTU	4I1G	4QKK	5DEK	5WSP	5WSS	5XK0
			6DWT	6DXJ								
143	$H\ 3$ or $P\ 3$	trigonal	332D	4RHD								
154	$P\ 3_1\ 2\ 1$		1IH4	1IH6	3SSF	5MVK	5MVL	5MVP	5MVQ	5MVT	6L75	
169	$P\ 6_1$	hexagonal	3IFF	4F4N	5XK1							
178	$P\ 6_1\ 2\ 2$		138D	1DNZ	260D	2B1B	327D	348D	349D	395D	396D	441D
			5FHL									

[a] Space group number and crystal system were obtained from the *International Tables for Crystallography*.[2]

[b] Space groups full names were obtained as written in PDB file ("REMARK 290 SYMMETRY OPERATORS FOR SPACE GROUP:")

5. Distribution of NMR models per PDB entry in NMR dataset

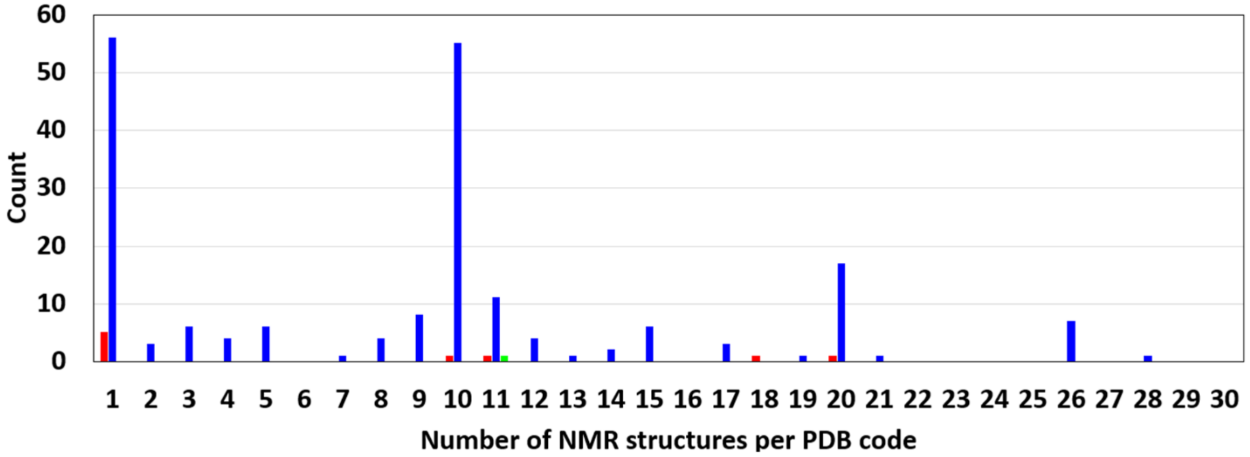

Supplementary Figure S3: Distribution of the number of models per PDB code (ensemble of NMR determined structures) in the NMR dataset for A-helix (red), B-helix (blue) and Z-helix (green).

6. Scatter matrices of nucleic acid torsions

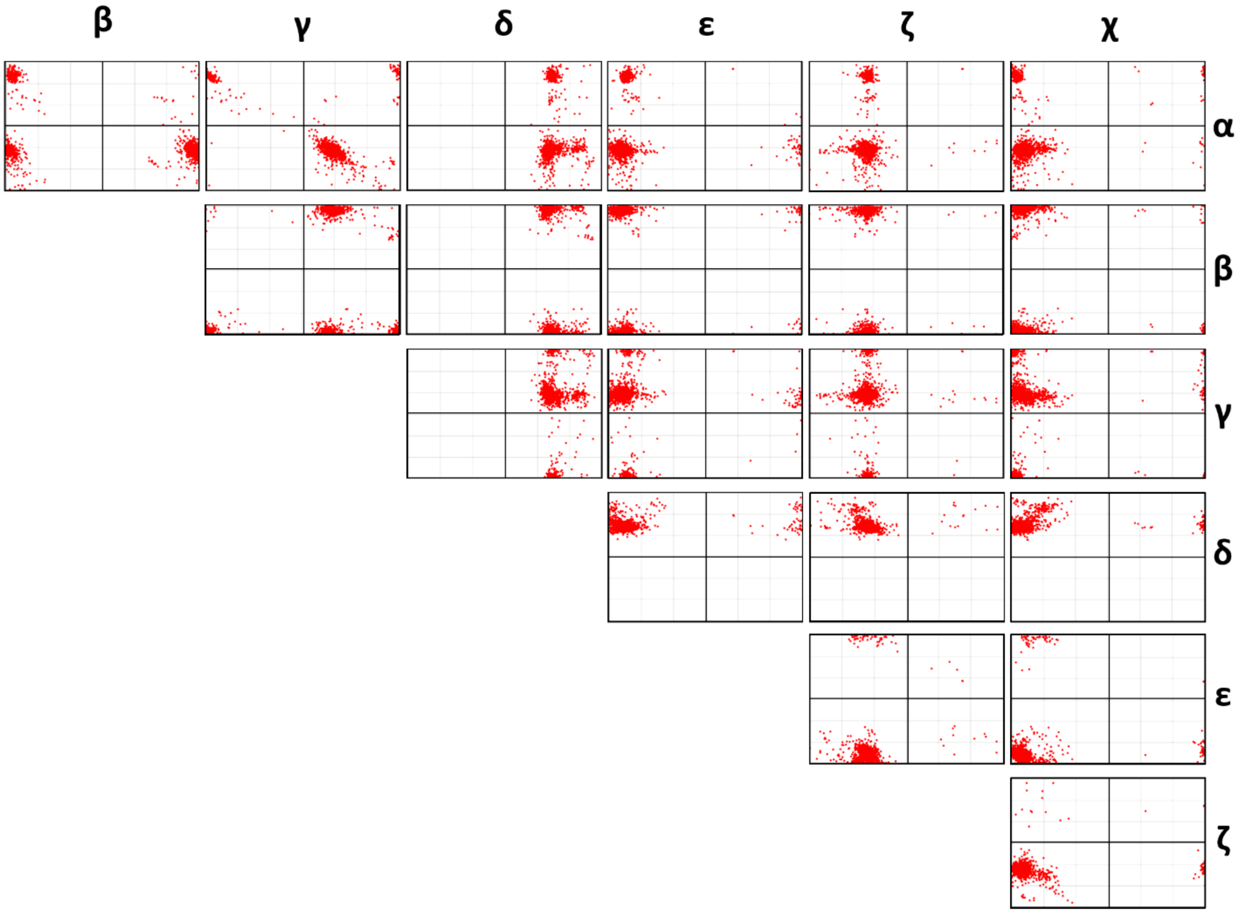

Supplementary Figure S4: Scatter matrix of nucleic acid torsions of A-helices from X-ray diffraction methods (resolution: 2 Å or less) obtained from the RCSB[3–5]. Both x- and y-axis scale from -180° to +180°, with 0° indicated by black reference lines. An x-axis label for each column of plots is given on top, a y-axis label for each row of plots is given on the right.

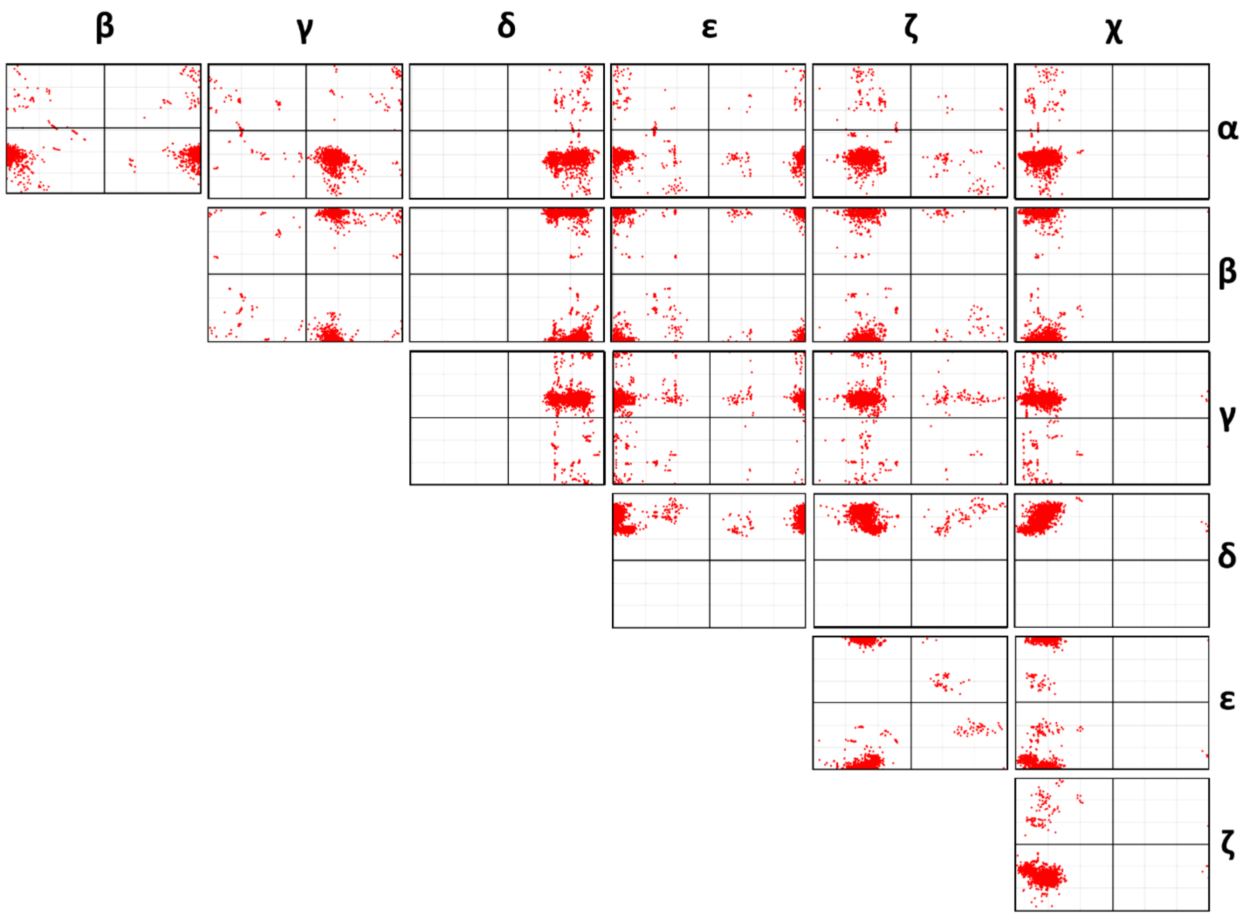

Supplementary Figure S5: Scatter matrix of nucleic acid torsions of A-helices from NMR solution methods obtained from the RCSB.[3–5] Both x- and y-axis scale from -180° to +180°, with 0° indicated by black reference lines. An x-axis label for each column of plots is given on top, a y-axis label for each row of plots is given on the right.

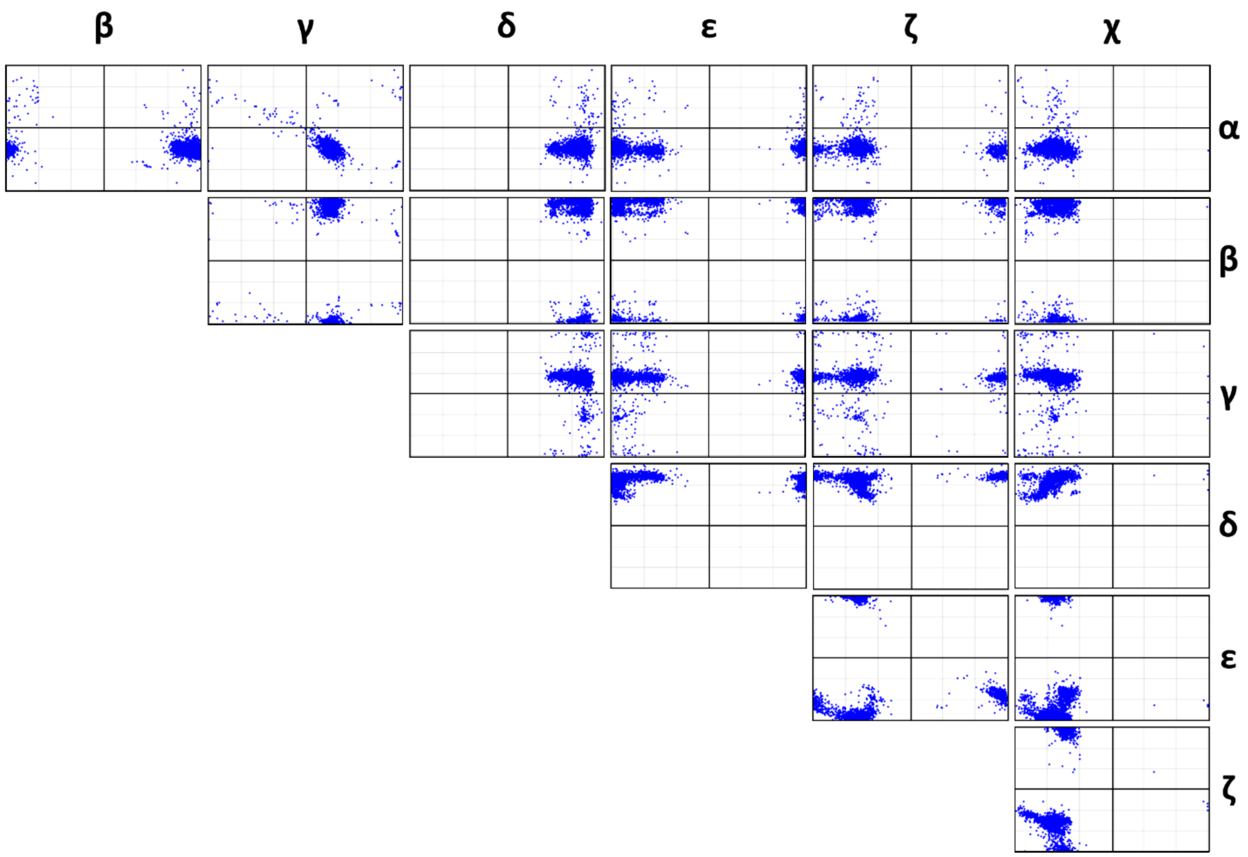

Supplementary Figure S6: Scatter matrix of nucleic acid torsions of B-helices from X-ray diffraction methods (resolution: 2 Å or less) obtained from the RCSB.[3–5]. Both x- and y-axis scale from -180° to +180°, with 0° indicated by black reference lines. An x-axis label for each column of plots is given on top, a y-axis label for each row of plots is given on the right.

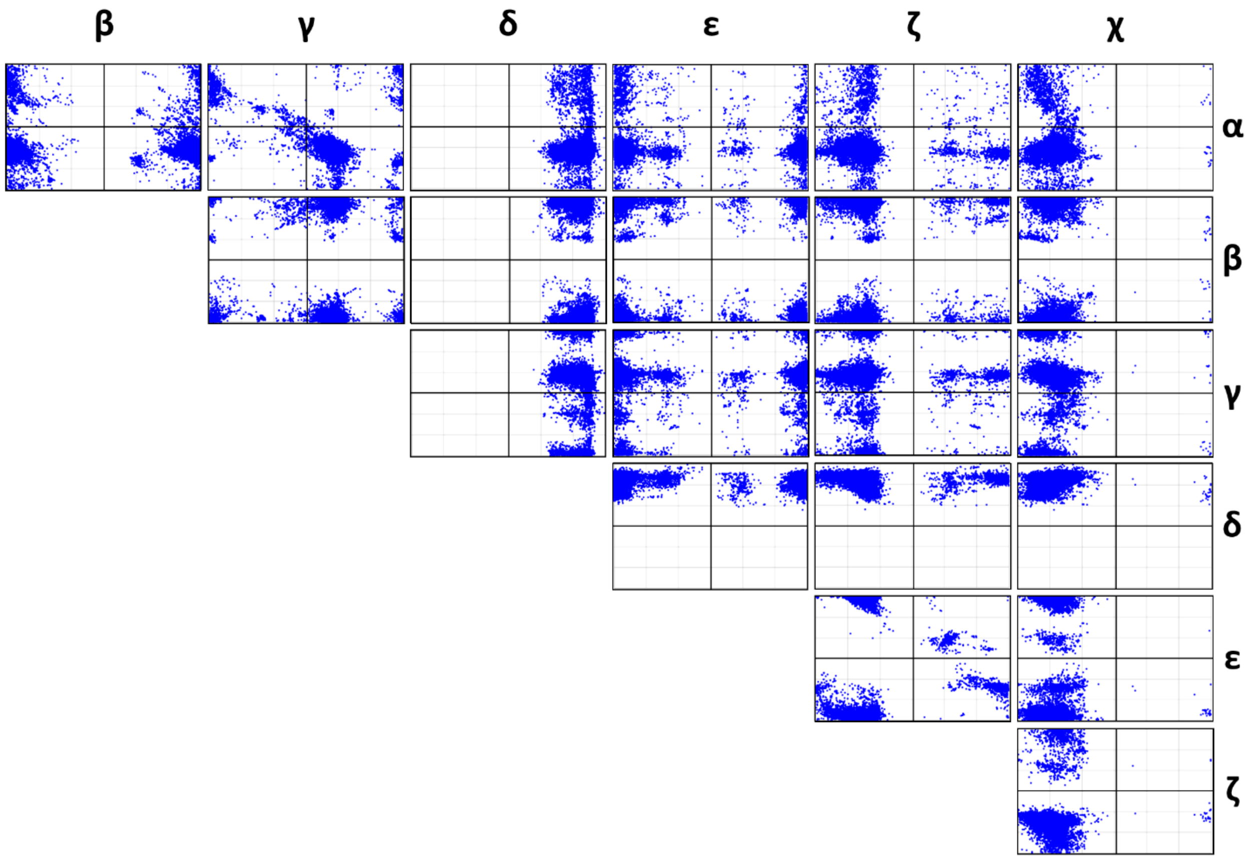

Supplementary Figure S7: Scatter matrix of nucleic acid torsions of B-helices from NMR solution methods obtained from the RCSB.[3–5]. Both x- and y-axis scale from -180° to +180°, with 0° indicated by black reference lines. An x-axis label for each column of plots is given on top, a y-axis label for each row of plots is given on the right.

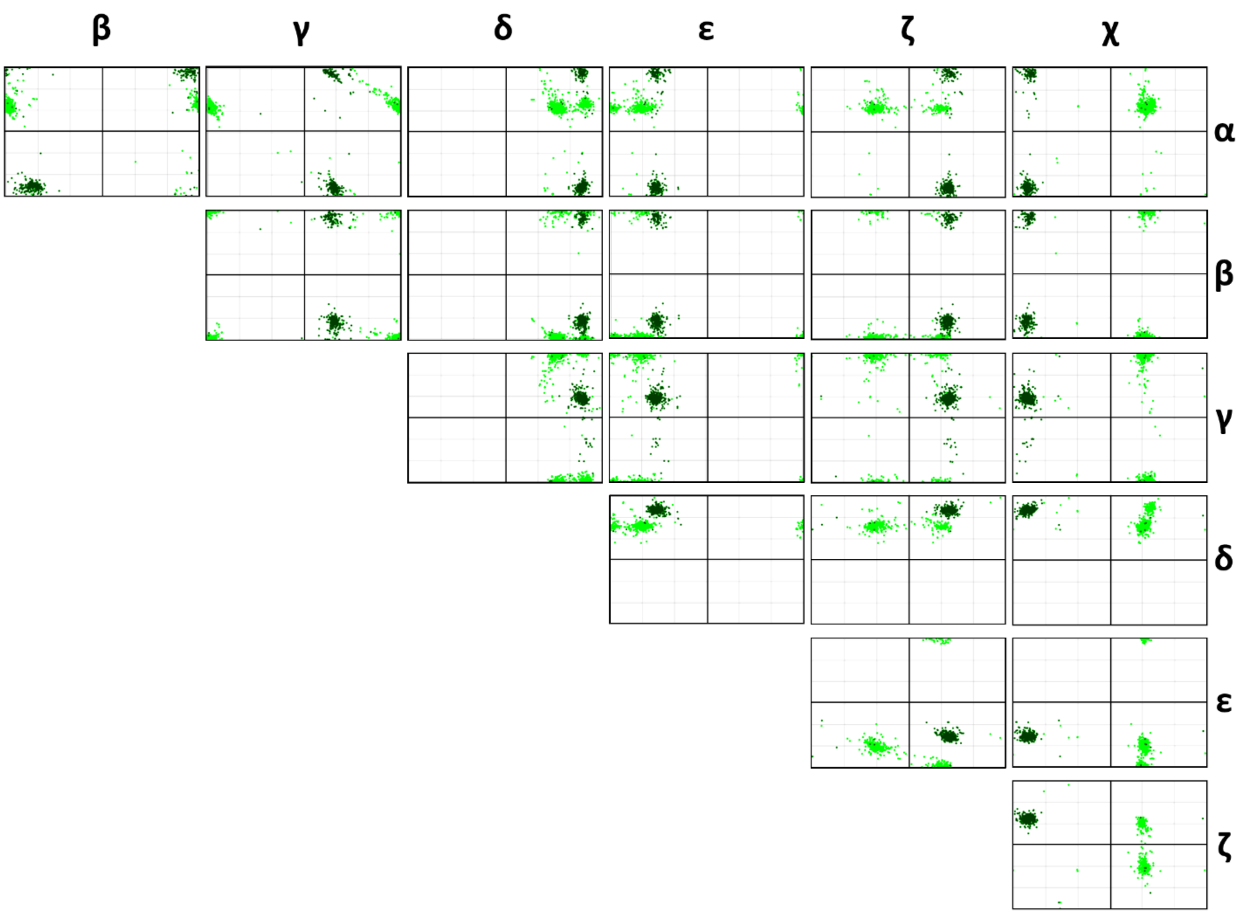

Supplementary Figure S8: Scatter matrix of nucleic acid torsions of Z-helices (separated into purine and pyrimidine; green and dark green, respectively) from X-ray diffraction methods (resolution: 2 Å or less) obtained from the RCSB.[3–5] Both x- and y-axis scale from -180° to +180°, with 0° indicated by black reference lines. An x-axis label for each column of plots is given on top, a y-axis label for each row of plots is given on the right.

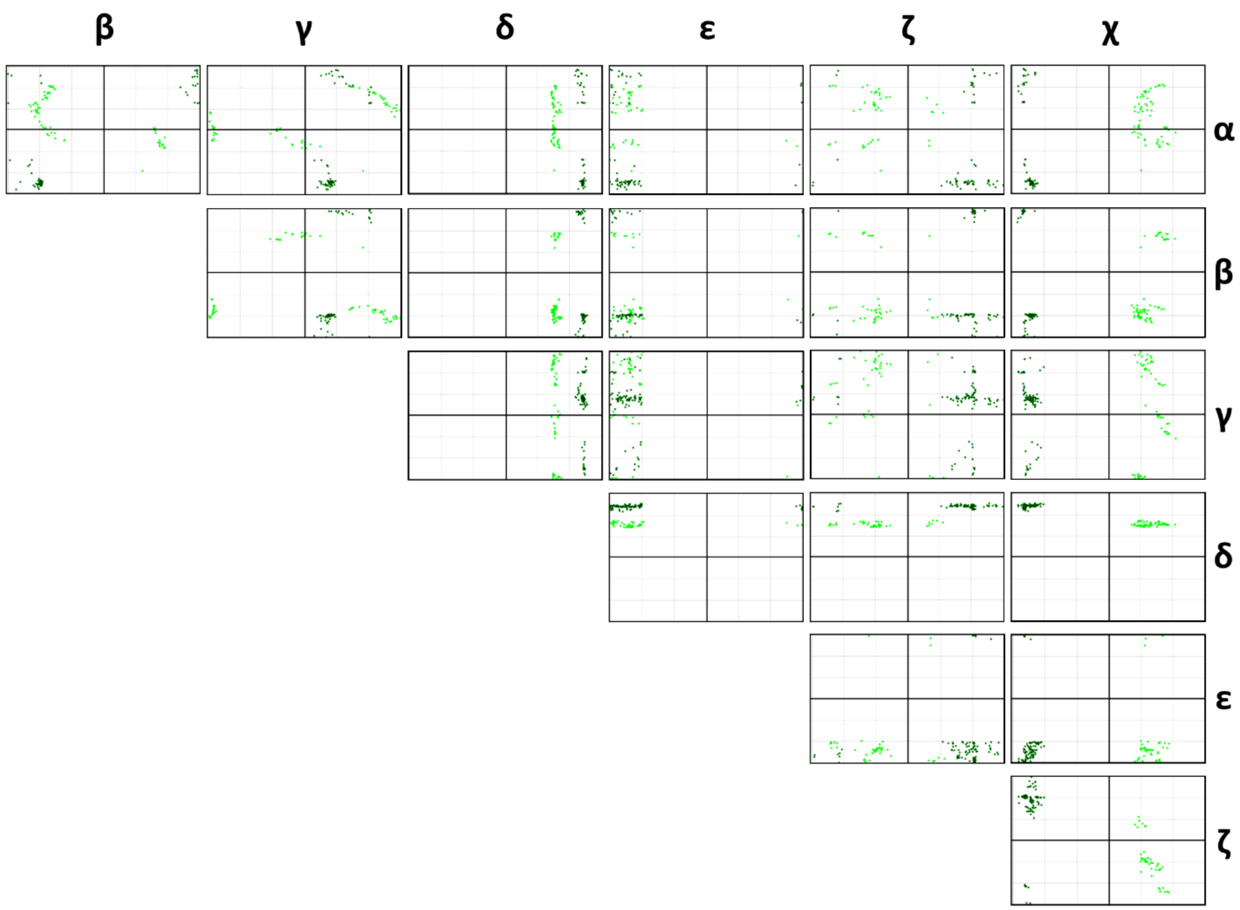

Supplementary Figure S9: Scatter matrix of nucleic acid torsions of Z-helices (separated into purine and pyrimidine; green and dark green, respectively) from NMR solution methods obtained from the RCSB.[3–5] Both x- and y-axis scale from -180° to +180°, with 0° indicated by black reference lines. An x-axis label for each column of plots is given on top, a y-axis label for each row of plots is given on the right.

7. Field histograms A- and B-Helix (NMR dataset)

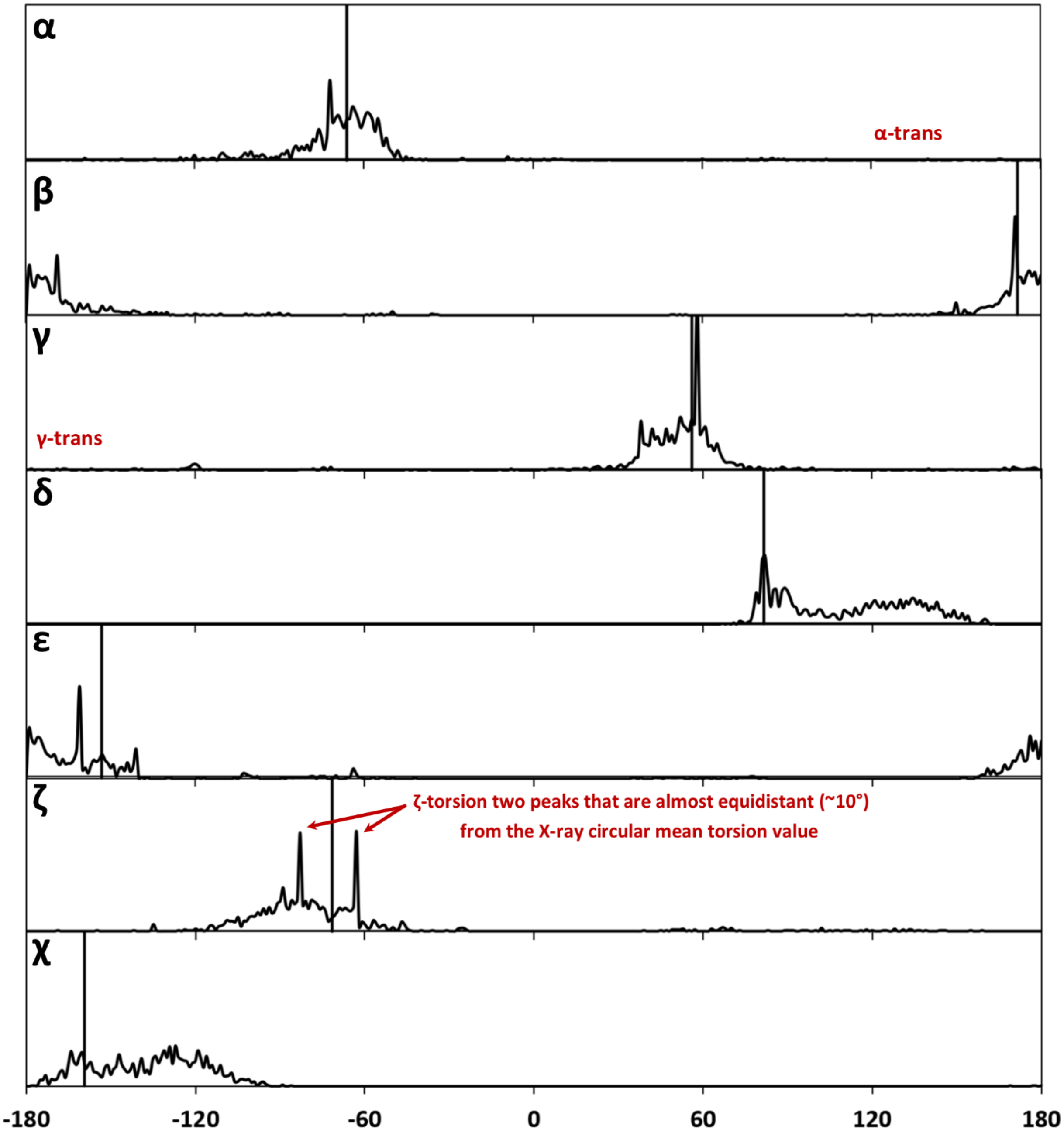

Supplementary Figure S10: Field histogram of NMR A-helix torsion angles (°). The x-axis is shared for each torsion and ranges from -180° to 180° and the y-axis is the count of the frequency of occurrence by torsion angle (scale: 0 to 200). The solid vertical line represents the angle peak for each torsion measured in this work from the X-ray A-helix structures. Regions associated with α-trans/γ-trans conformation as well as ζ-torsion peaks are identified.

Supplementary Table S7: NMR A-form structures and reported experimental conditions

PDB	NMR conditions [a]
124D[6]	50 mM sodium phosphate (pH 7.0), 100 mM NaCl, 99.996% D_2O
140D[7,8]	50 mM NaCl (pH 7.0; no buffer), ~1.8 mM duplex DNA, 99.995% D_2O (additional sample in 90% H_2O/10% D_2O)
142D[7,8]	50 mM NaCl (pH 7.0; no buffer), ~1.8 mM duplex DNA, 99.995% D_2O (additional sample in 90% H_2O/10% D_2O)
1D68[9]	20 mM sodium phosphate (pH 7.0), 200 mM NaCl, 99.996% D_2O; NMR at 30 °C
1DHH[10]	50 mM sodium phosphate (pH 6.95), 100 mM NaCl, 3 mM EDTA, 3 mM duplex DNA, D_2O (298 K) or H_2O (288 K)
1L3M[11]	20 mM sodium phosphate (pH 7.0), 200 mM NaCl, 0.05 mM EDTA, 90% H_2O /10% D_2O; 99.996% D_2O for non-exchangeable proton studies
2LAR[12]	10 mM sodium phosphate, 50 mM NaCl, 0.1 mM EDTA; 80 µM duplex in 90% H_2O /10% D_2O (pH 6.2) or ~1.0 mM duplex in D_2O (pH* 6.6); NMR at 298 K
2LB4[12]	10 mM sodium phosphate, 50 mM NaCl, 0.1 mM EDTA; 80 µM duplex in 90% H_2O /10% D_2O (pH 6.2) or ~1.0 mM duplex in D_2O (pH* 6.6); NMR at 298 K
2M1V[13]	0.5-0.8 mM RNA-DNA hybrid, 110 mM KCl, 10 µM EDTA; 100% D_2O or 90% H_2O /10% D_2O; pH 6.8 (pD 6.8 in D_2O)
2M84[14]	25 mM sodium phosphate (pH 7.0), 100 mM NaCl; 100% D_2O or 90% H_2O /10% D_2O
2M8A[14]	25 mM sodium phosphate (pH 7.0), 100 mM NaCl; 100% D_2O or 90% H_2O /10% D_2O
2O7W[15]	10 mM sodium phosphate (pH 6.5), 0.2 mM EDTA, 2.5-2.7 mM duplex DNA; 100% D_2O or 90% H_2O /10% D_2O
5B81	350 µM duplex DNA, 50 mM NaCl, pH 7.2; 95% H_2O /5% D_2O; NMR at 288 K
5TGG[16]	1.0 mM duplex, 50 mM sodium acetate-d_3 (pH 4.0); 100% D_2O or 90% H_2O /10% D_2O
6GMY[17]	0.5 mM duplex, 10 mM sodium phosphate (pH 7.05), 50 mM NaCl; D_2O (283, 298 K) or 90% H_2O /10% D_2O (283 K)
7NBL[18]	25 mM potassium phosphate (pH 7.0), 100 mM NaCl; D_2O or 90% H_2O /10% D_2O; NMR at 25 °C (D_2O) or 5 °C (H_2O)
7NEJ[18]	25 mM potassium phosphate (pH 7.0), 100 mM NaCl; D_2O or 90% H_2O /10% D_2O; NMR at 25 °C (D_2O) or 5 °C (H_2O)

[a] Experimental solution conditions were obtained from the associated primary publications; for PDB code 5B81, conditions were obtained from the PDB experimental metadata.

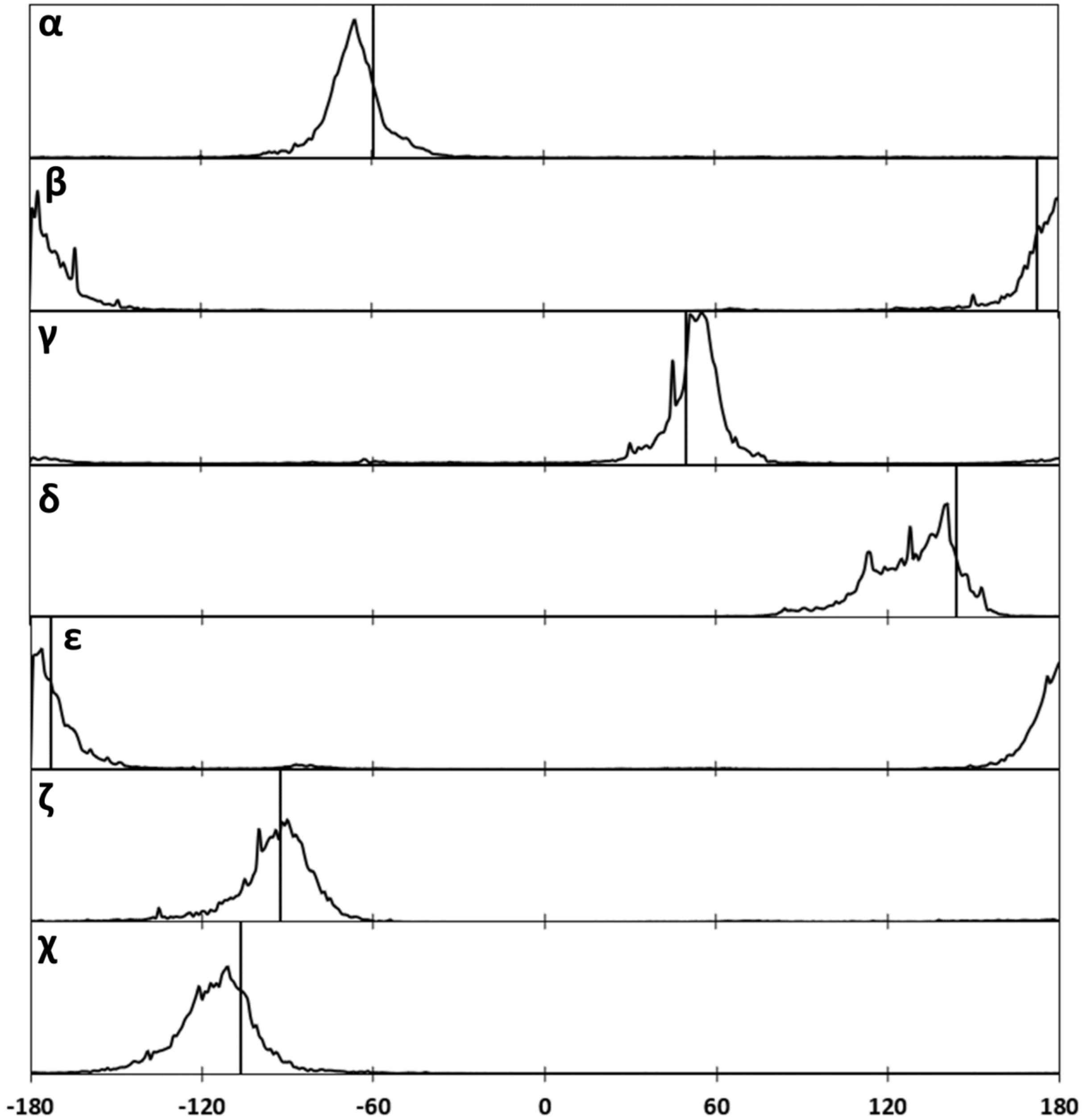

Supplementary Figure S11: Field histogram of NMR B-helix torsion angles (°). The x-axis is shared for each torsion and ranges from -180° to 180° and the y-axis is the count of the frequency of occurrence by torsion angle (scale: 0 to 2,000). The solid vertical line represents the angle peak for each torsion measured in this work from the X-ray B-helix structures.

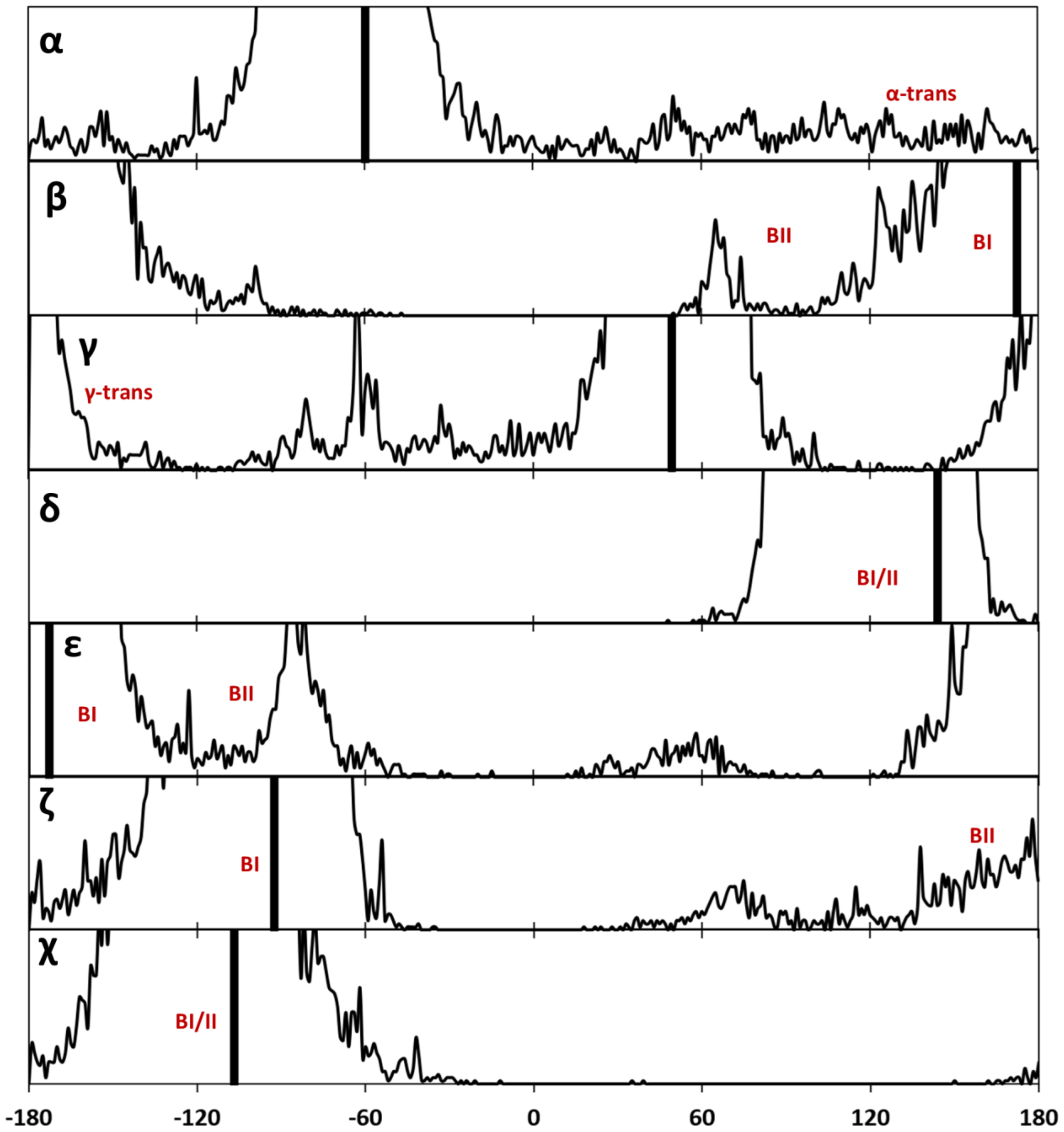

Supplementary Figure S12: Field histogram of NMR B-helix torsion angles (°). The x-axis is shared for each torsion and ranges from -180° to 180° and the y-axis is the count of the frequency of occurrence by torsion angle (scale: 0 to 50). The solid vertical line represents the angle peak for each torsion measured in this work from the X-ray B-helix structures. Peaks associated with BI-/BII-DNA as well as regions associated with α-trans/γ-trans conformations are identified.

8. Modeled 1BNA

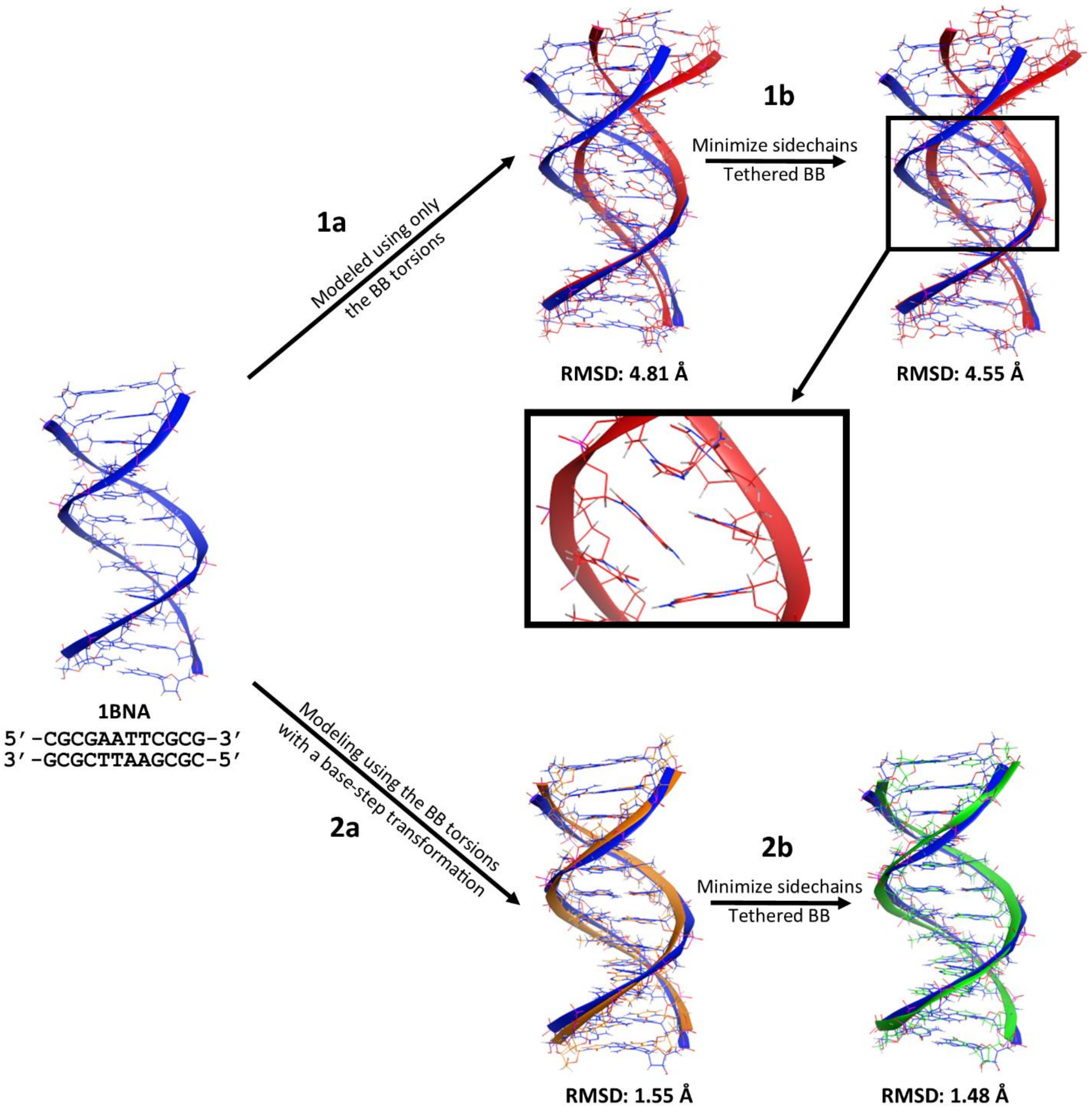

Supplementary Figure S13: Modeling the Dickerson-Drew B-DNA dodecamer sequence (PDB ID: 1BNA[1]) using two different modeling approaches. The crystal structure (1BNA) is shown in blue and the modeled helices are shown in red, orange and green which have the worst to best levels of accuracy, respectively. Pathway 1 approach (above) uses only the backbone torsions. Pathway 2 approach (below) uses the backbone torsions with a base-step transformation. DNA backbone atoms (P, O5', C5', C4', C3', O3', C1', C2', and O4') used for superposition/measuring backbone RMSD.

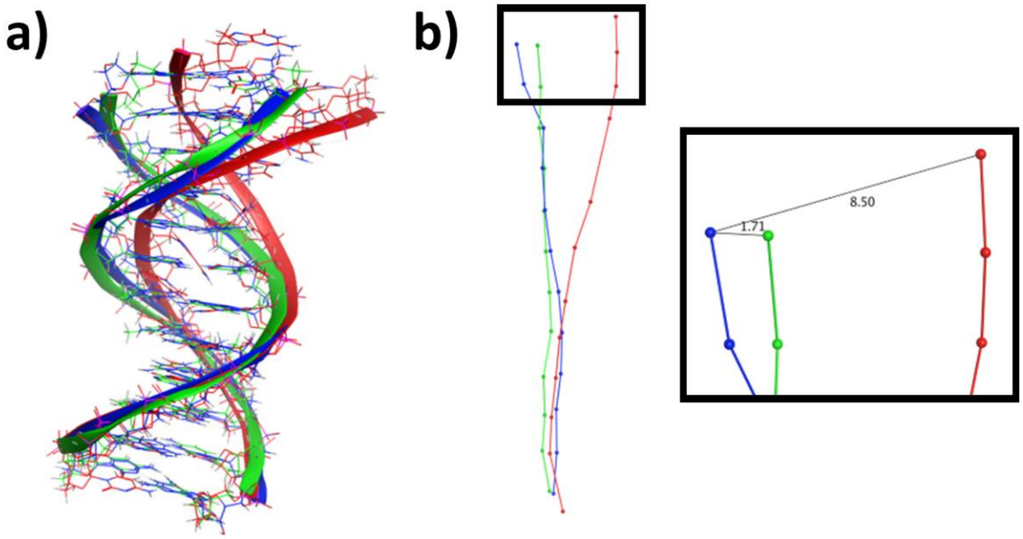

Supplementary Figure S14: Deviation of helical axis in modeled Dickerson-Drew B-DNA dodecamer sequence (PDB ID: 1BNA[1]) using two different modeling approaches. (a) Superposition (DNA backbone atoms) of X-ray (blue), pathway 1 modeling (red) and pathway 2 modeling (green) structures (see Supplementary Figure S13 for pathway descriptions). (b) Rendering of base-pair origin[19] for each structure to highlight the axis displacement of each model with respect to the X-ray structure. Distance shown in Å. DNA backbone atoms (P, O5', C5', C4', C3', O3', C1', C2', and O4') used for superposition/measuring backbone RMSD.

9. Motif Grafting and Model Construction Protocol

The model of 6HMI[20] (**Figure 13c**) was generated by first constructing the canonical A-helix portion, followed by grafting on a single bulged A motif by superimposing the terminal base pair of the motif fragment onto the ends of the A-helix framework. Using the RNA 3D Motif Atlas[21] (internal loops; Release 3.86), a motif template (PDB ID: 7DLZ[22]) was selected from 89 instances for illustrative purposes. The backbone RMSD (relative to the experimental structure) of the A-helix section and the bulged A motif were 2.66 Å and 3.25 Å, respectively, with an overall backbone RMSD of 2.95 Å.

The model of 6HMO[20] (**Figure 13d**) was generated in a similar manner by constructing the canonical A-helix portion, followed by grafting a single stack-bend motif *via* superposition of the terminal base pair onto the A-helix framework. Using the RNA 3D Motif Atlas[21] (internal loops; Release 3.86), a motif template (PDB ID: 4LFB[23]) was selected from 26 instances for illustrative purposes. The backbone RMSD (relative to the experimental structure) of the A-helix section and the stack-bend motif were 1.77 Å and 1.61 Å, respectively, with an overall backbone RMSD of 1.69 Å.

The ligand (SMN-C5) was positioned and optimized within the modelled binding pocket, achieving an RMSD of 2.30 Å relative to the experimental position (heavy atoms only). The high structural similarity of the stack-bend motif suggests that alternative construction strategies, such as extending the A-helix, introducing a strand break to remove the additional nucleotide, and rejoining the chains, may also be viable. Overall, the models of the *apo* and *holo* U1 snRNA:E7 5′-splice site are sensible, with both exhibiting an overall backbone RMSD of less than 3 Å. However, further improvements can be achieved by optimizing the selection of grafted motifs and anchor base-pairs.

One practical approach, among many, for leveraging such reference models is outlined here for illustrative purposes. Motif identification and reconstruction may proceed by first superposing the backbone atoms of anchor base pairs from library fragments onto a canonical framework (e.g. A-helix) to assess conformational similarity. Candidates exceeding a predefined maximum anchor RMSD can be discarded, while acceptable motifs may be grafted by rotation and translation into the same frame of reference as the framework. Additional evaluations may include assessment of sequence compatibility, quality of the join, steric clashes with the surrounding environment, and whether similar motif conformations have been observed previously. Adjustment of the anchor RMSD threshold may further refine the balance between selectivity and coverage.

10. Additional references

(1) Drew, H. R.; Wing, R. M.; Takano, T.; Broka, C.; Tanaka, S.; Itakura, K.; Dickerson, R. E. Structure of a B-DNA Dodecamer: Conformation and Dynamics. *Proceedings of the National Academy of Sciences* **1981**, *78* (4), 2179–2183. https://doi.org/10.1073/pnas.78.4.2179.

(2) *International Tables for Crystallography*; Hahn, T., Shmueli, U., Wilson, A. J. C., International Union of Crystallography, Eds.; D. Reidel Pub. Co. ; Sold and distributed in the U.S.A. and Canada by Kluwer Academic Publishers Group: Dordrecht, Holland ; Boston, U.S.A. : Hingham, MA, 1984.

(3) Crystallography: Protein Data Bank. *Nature New Biology* **1971**, *233* (42), 223–223. https://doi.org/10.1038/newbio233223b0.

(4) Berman, H. M.; Westbrook, J.; Feng, Z.; Gilliland, G.; Bhat, T. N.; Weissig, H.; Shindyalov, I. N.; Bourne, P. E. The Protein Data Bank. *Nucleic Acids Research* **2000**, *28* (1), 235–242. https://doi.org/10.1093/nar/28.1.235.

(5) Bank, R. P. D. *RCSB PDB*. https://www.rcsb.org/ (accessed 2024-01-13).

(6) Fedoroff, O. Yu.; Salazar, M.; Reid, B. R. Structure of a DNA : RNA Hybrid Duplex: Why RNase H Does Not Cleave Pure RNA. *Journal of Molecular Biology* **1993**, *233* (3), 509–523. https://doi.org/10.1006/jmbi.1993.1528.

(7) Mujeeb, A.; Kerwin, S. M.; Egan, W.; Kenyon, G. L.; James, T. L. A Potential Gene Target in HIV-1: Rationale, Selection of a Conserved Sequence, and Determination of NMR Distance and Torsion Angle Constraints. *Biochemistry* **1992**, *31* (39), 9325–9338. https://doi.org/10.1021/bi00154a002.

(8) Mujeeb, A.; Kerwin, S. M.; Kenyon, G. L.; James, T. L. Solution Structure of a Conserved DNA Sequence from the HIV-1 Genome: Restrained Molecular Dynamics Simulation with

Distance and Torsion Angle Restraints Derived from Two-Dimensional NMR Spectra. *Biochemistry* **1993**, *32* (49), 13419–13431. https://doi.org/10.1021/bi00212a007.

(9) Cheng, J.-W.; Chou, S.-H.; Salazar, M.; Reid, B. R. Solution Structure of [d(GCGTATACGC)]2. *Journal of Molecular Biology* **1992**, *228* (1), 118–137. https://doi.org/10.1016/0022-2836(92)90496-7.

(10) Nishizaki, T.; Iwai, S.; Ohkubo, T.; Kojima, C.; Nakamura, H.; Kyogoku, Y.; Ohtsuka, E. Solution Structures of DNA Duplexes Containing a DNA·RNA Hybrid Region, d(GG)r(AGAU)d(GAC)·d(GTCATCTCC) and d(GGAGA)r(UGAC)·d(GTCATCTCC),. *Biochemistry* **1996**, *35* (13), 4016–4025. https://doi.org/10.1021/bi9519821.

(11) Tsao, Y.-P.; Wang, L.-Y.; Hsu, S.-T.; Jain, M. L.; Chou, S.-H.; Huang, W.-C.; Cheng, J.-W. The Solution Structure of [d(CGC)r(Amamam)d(TTTGCG)]2. *J Biomol NMR* **2001**, *21* (3), 209–220. https://doi.org/10.1023/A:1012924932513.

(12) Johnson, C. N.; Spring, A. M.; Sergueev, D.; Shaw, B. R.; Germann, M. W. Structural Basis of the RNase H1 Activity on Stereo Regular Borano Phosphonate DNA/RNA Hybrids. *Biochemistry* **2011**, *50* (19), 3903–3912. https://doi.org/10.1021/bi200083d.

(13) Skilandat, M.; Sigel, R. K. O. The Role of Mg(II) in DNA Cleavage Site Recognition in Group II Intron Ribozymes: SOLUTION STRUCTURE AND METAL ION BINDING SITES OF THE RNA·DNA COMPLEX *. *Journal of Biological Chemistry* **2014**, *289* (30), 20650–20663. https://doi.org/10.1074/jbc.M113.542381.

(14) Martin-Pintado, N.; Deleavey, G. F.; Portella, G.; Campos-Olivas, R.; Orozco, M.; Damha, M. J.; González, C. Backbone FC□H⋯O Hydrogen Bonds in 2′F-Substituted Nucleic Acids. *Angewandte Chemie International Edition* **2013**, *52* (46), 12065–12068. https://doi.org/10.1002/anie.201305710.

(15) Chen, J.; Dupradeau, F.-Y.; Case, D. A.; Turner, C. J.; Stubbe, J. DNA Oligonucleotides with A, T, G or C Opposite an Abasic Site: Structure and Dynamics. *Nucleic Acids Res* **2008**, *36* (1), 253–262. https://doi.org/10.1093/nar/gkm622.

(16) Xie, R.; Xiao, G.; Li, W.; Luochen, W.; Butt, M.; Que, Y.; Li, X.; Li, Y.; Sun, L.; Chen, G. Late-Stage Adenine N6-Alkylation of Nucleos(t)Ides and Oligonucleotides via Photoredox and Copper Co-Catalytic Decarboxylative C(Sp3)–N Coupling. *Nat Commun* **2025**, *17* (1), 1090. https://doi.org/10.1038/s41467-025-67851-w.

(17) Istrate, A.; Johannsen, S.; Istrate, A.; Sigel, R. K. O.; Leumann, C. J. NMR Solution Structure of Tricyclo-DNA Containing Duplexes: Insight into Enhanced Thermal Stability and Nuclease Resistance. *Nucleic Acids Res* **2019**, *47* (9), 4872–4882. https://doi.org/10.1093/nar/gkz197.

(18) Cabrero, C.; Martín-Pintado, N.; Mazzini, S.; Gargallo, R.; Eritja, R.; Aviñó, A.; González, C. Structural Effects of Incorporation of 2'-Deoxy-2'2'-Difluorodeoxycytidine (Gemcitabine) in A- and B-Form Duplexes. *Chemistry – A European Journal* **2021**, *27* (26), 7351–7355. https://doi.org/10.1002/chem.202100503.

(19) Olson, W. K.; Bansal, M.; Burley, S. K.; Dickerson, R. E.; Gerstein, M.; Harvey, S. C.; Heinemann, U.; Lu, X. J.; Neidle, S.; Shakked, Z.; Sklenar, H.; Suzuki, M.; Tung, C. S.; Westhof, E.; Wolberger, C.; Berman, H. M. A Standard Reference Frame for the Description of Nucleic Acid Base-Pair Geometry. *Journal of Molecular Biology* **2001**, *313* (1), 229–237. https://doi.org/10.1006/jmbi.2001.4987.

(20) Campagne, S.; Boigner, S.; Rüdisser, S.; Moursy, A.; Gillioz, L.; Knörlein, A.; Hall, J.; Ratni, H.; Cléry, A.; Allain, F. H.-T. Structural Basis of a Small Molecule Targeting RNA for

a Specific Splicing Correction. *Nat Chem Biol* **2019**, *15* (12), 1191–1198. https://doi.org/10.1038/s41589-019-0384-5.

(21) Petrov, A. I.; Zirbel, C. L.; Leontis, N. B. Automated Classification of RNA 3D Motifs and the RNA 3D Motif Atlas. *RNA* **2013**, *19* (10), 1327–1340. https://doi.org/10.1261/rna.039438.113.

(22) Jiang, H.; Gao, Y.; Zhang, L.; Chen, D.; Gan, J.; Murchie, A. I. H. The Identification and Characterization of a Selected SAM-Dependent Methyltransferase Ribozyme That Is Present in Natural Sequences. *Nat Catal* **2021**, *4* (10), 872–881. https://doi.org/10.1038/s41929-021-00685-z.

(23) Demirci, H.; Belardinelli, R.; Carr, J.; Murphy IV, F.; Jogl, G.; Dahlberg, A. E.; Gregory, S. T. Crystal Structure of 30S Ribosomal Subunit from Thermus Thermophilus. https://doi.org/https://doi.org/10.2210/pdb4LFB/pdb.
